# Brassinosteroid gene regulatory networks at cellular resolution in the *Arabidopsis* root

**DOI:** 10.1126/science.adf4721

**Published:** 2023-03-31

**Authors:** Trevor M Nolan, Nemanja Vukašinović, Che-Wei Hsu, Jingyuan Zhang, Isabelle Vanhoutte, Rachel Shahan, Isaiah W Taylor, Laura Greenstreet, Matthieu Heitz, Anton Afanassiev, Ping Wang, Pablo Szekely, Aiden Brosnan, Yanhai Yin, Geoffrey Schiebinger, Uwe Ohler, Eugenia Russinova, Philip N Benfey

**Affiliations:** 1 Department of Biology, Duke University; Durham, USA.; 2 Department of Plant Biotechnology and Bioinformatics, Ghent University; Ghent, Belgium.; 3 Center for Plant Systems Biology, VIB; Ghent, Belgium.; 4 Department of Biology, Humboldt Universitat zu Berlin; Berlin, Germany.; 5 The Berlin Institute for Medical Systems Biology, Max Delbruck Center for Molecular Medicine; Berlin, Germany.; 6 University of British Columbia, Department of Mathematics; Vancouver, Canada.; 7 Department of Genetics, Development and Cell Biology, Iowa State University; Ames, USA.; 8 Department of Computer Science, Humboldt Universitat zu Berlin; Berlin, Germany.; 9 Howard Hughes Medical Institute, Duke University; Durham, USA.

## Abstract

Brassinosteroids are plant steroid hormones that regulate diverse processes such as cell division and cell elongation through gene regulatory networks that vary in space and time. By using time-series single-cell RNA-sequencing to profile brassinosteroid-responsive gene expression specific to different cell types and developmental stages of the *Arabidopsis* root, we identified the elongating cortex as a site where brassinosteroids trigger a shift from proliferation to elongation associated with increased expression of cell wall-related genes. Our analysis revealed *HOMEOBOX FROM ARABIDOPSIS THALIANA 7* (*HAT7*) and *GT-2-LIKE 1* (*GTL1*) as brassinosteroid-responsive transcription factors that regulate cortex cell elongation. These results establish the cortex as a site of brassinosteroid-mediated growth and unveil a brassinosteroid signaling network regulating the transition from proliferation to elongation, illuminating aspects of spatiotemporal hormone response.

## One-Sentence Summary

Single-cell RNA-sequencing identifies the cortex as a site of brassinosteroid-dependent growth in *Arabidopsis* roots.

During development, cells pass through different states as they acquire identities and progress toward end-stage differentiation (1). Gene regulatory networks (GRNs) control this progression and must be tuned according to developmental stage, cell identity, and environmental conditions (2–4). Signaling molecules such as hormones are central players in coordinating these networks, but it has been challenging to disentangle how cell identities, developmental states, and hormone responses influence one another. Technological advances in single-cell RNA-sequencing (scRNA-seq) (2, 5) and tissue-specific gene manipulations (6, 7) make it possible to address this challenge using the *Arabidopsis* root as a model system.

Brassinosteroids are a group of plant steroid hormones that affect cell division and cell elongation during root growth (8–12). Brassinosteroids are sensed at the plasma membrane by BRASSINOSTEROID INSENSITIVE 1 (BRI1) family receptors (13–16), initiating signal transduction events that activate BES1 and BZR1 family transcription factors to control thousands of genes (17–20). The brassinosteroid GRN is typically represented singularly without consideration of cell specificity (20–23), even though brassinosteroids lead to different responses depending on the developmental context (24–28).

By profiling brassinosteroid responses across the cell types and developmental stages of the root using scRNA-seq, we discovered that brassinosteroids affect gene expression in the elongating cortex. Reconstruction of cortex trajectories over a scRNA-seq time course showed that brassinosteroids trigger a shift from proliferation to elongation, which is associated with upregulation of cell wall-related genes. Loss of brassinosteroid signaling in the cortex using tissue-specific CRISPR reduced cell elongation. Our time-course data allowed us to propose brassinosteroid-responsive GRNs, which led to the identification of HAT7 and GTL1 as validated regulators of brassinosteroid response in the elongating cortex. These datasets represent 210,856 single-cell transcriptomes, providing a high-resolution view of brassinosteroid-mediated GRNs.

## Results:

### scRNA-seq reveals spatiotemporal brassinosteroid responses

To investigate spatiotemporal brassinosteroid responses in the *Arabidopsis* root, we used a sensitized system, which involved inhibiting brassinosteroid biosynthesis using Brassinazole (BRZ) (29), then reactivating signaling with Brassinolide (BL), the most active brassinosteroid (10, 21, 30) (fig. S1). We treated 7-day-old primary roots for 2 hours with BL or a corresponding mock BRZ control and performed scRNA-seq on protoplasts isolated from three biological replicates of 0.5 cm root tips (containing meristem, elongation, and early differentiation zones) using the 10X Genomics Chromium system (Methods).

To annotate cell types and developmental stages, we performed label transfer based on our single-cell atlas of the *Arabidopsis* root (31). We distinguished between two domains of the meristem: the proliferation domain, where cells have a high probability of dividing, and the transition domain, where cells divide less frequently but have not yet begun rapid expansion (fig. S2A–D and Data S2) (32, 33).

After data integration, the 11 major cell types and eight developmental stages identified were logically arranged in 2D uniform manifold approximation and projection (UMAP) space as previously described for root datasets (fig. S3A–B) (31, 34). Marker genes characteristic of cell types and developmental stages remained enriched, suggesting that although brassinosteroids alter the expression of thousands of genes, cell identities can be successfully aligned through integration (fig. S3C).

**Fig. 1.**
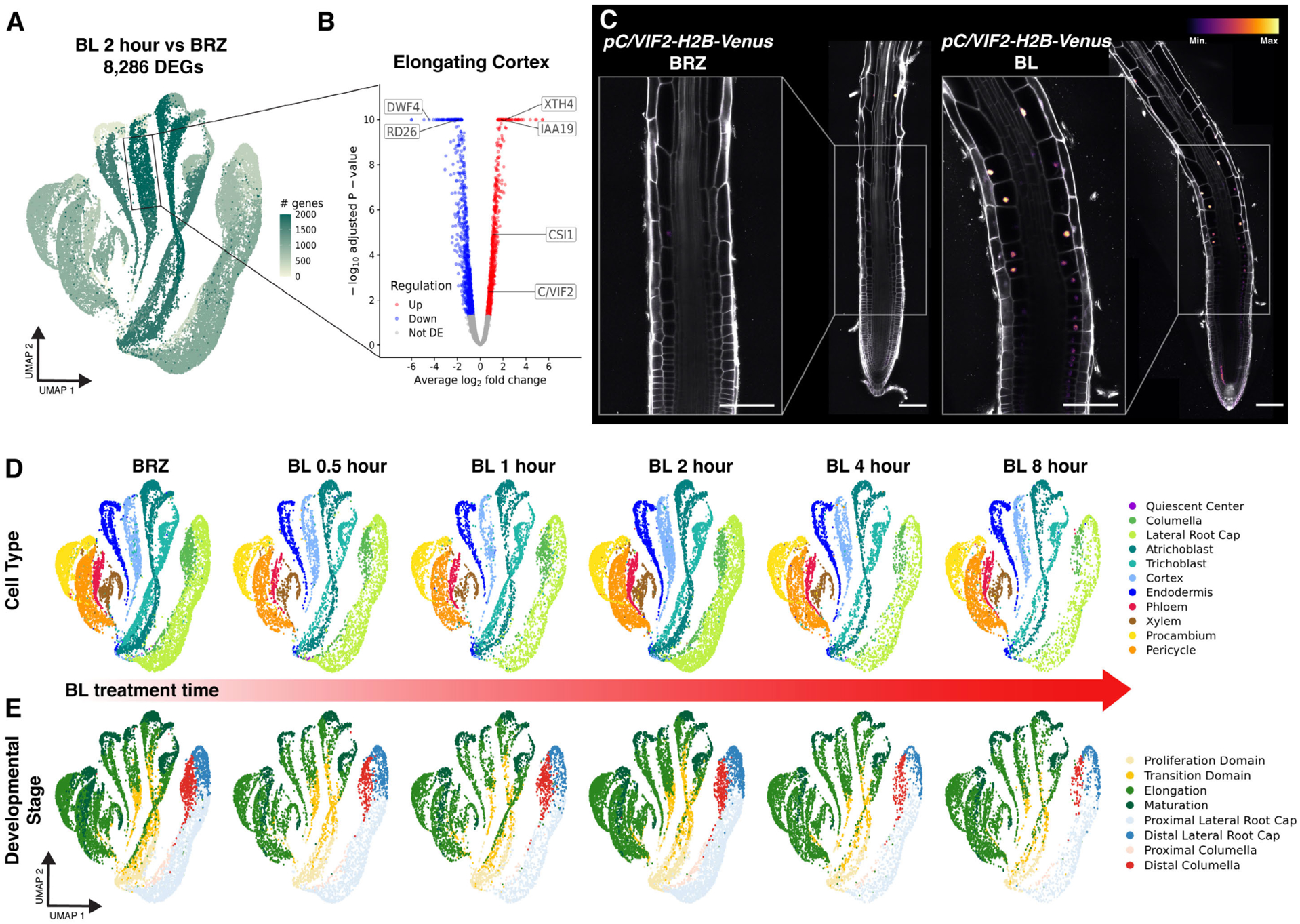
scRNA-seq identifies brassinosteroid-induction of cell wall-related genes in the cortex. **(A)** Spatiotemporal response to 2-hour BL treatment vs BRZ control among each combination of cell type and developmental stage of the *Arabidopsis* root. Color on UMAP projection indicates the number of differentially expressed genes (DEGs). **(B)** Volcano plot of BL DEGs in the elongating cortex. Color indicates the direction of regulation. Known markers of brassinosteroid response including *DWF4, RD26, XTH4*, and *IAA19* are indicated. *C/VIF2* and *CSI1* (described in this study) are also indicated. **(C)** *pC/VIF2-H2B-Venus* reporter grown on 1 μM BRZ for 7 days and transferred to 1 μM BRZ or 100 nM BL for 4 hours. Inset shows *C/VIF2* signals in the elongating cortex that increase with BL treatment. Propidium iodide-staining is shown in grey, with the color gradient indicating relative *C/VIF2-H2B-Venus* levels. Scale bars, 100 μm. **(D-E)** UMAP of BL treatment scRNA-seq time course. Mock BRZ control represents time 0. Colors indicate cell type (D) or developmental stage annotation (E).

Previous studies have profiled brassinosteroid-responsive gene expression in bulk tissue or in a handful of cell types, conflating cell type and developmental stage (23, 27, 35). To obtain a better spatiotemporal resolution, we performed differential expression analysis for each combination of cell type and developmental stage using pseudobulk expression profiles (see methods). We identified 8,286 differentially expressed genes (DEGs; Fold-change >1.5, False discovery rate <0.05; Fig. 1A and fig. S4A–B), which were enriched in BES1 and BZR1 targets and had significant overlap with previously identified brassinosteroid-regulated genes (fig. S4C and Data S3).

We found that 37% of DEGs were altered in a single cell type/developmental stage and more than 82% were differentially expressed in 5 or fewer cell type/developmental stage combinations (fig. S4B). This indicates that although brassinosteroids broadly influence gene expression, they modulate distinct sets of genes in different spatiotemporal contexts.

Among the tissues with many DEGs was the epidermis, as previously described (9, 12, 24, 27, 35). Atrichoblasts, or non-hair cells in the epidermis were particularly affected, showing changes across both the meristem and elongation zone. Our data also indicated that brassinosteroids influence gene expression in the cortex, especially in the elongation zone (Fig. 1A and fig. S4A). The cortex has been linked to plant environmental interactions, including response to water limitation (36, 37) and hydrotropism (38, 39). However, it is unknown how brassinosteroids modulate gene expression in this tissue or what processes are affected. To address these questions, we focused on brassinosteroid-mediated gene expression in the elongating cortex.

### Brassinosteroids induce cell wall-related genes in the elongating cortex

We found that brassinosteroid treatment led to 967 up-regulated genes and 1,156 down-regulated genes in the elongating cortex (Fig. 1B and fig. S4A). Gene ontology (GO) analysis indicated the up-regulated genes were enriched for genes related to “cell wall organization or biogenesis”, which is intriguing given the role of brassinosteroids in promoting cell elongation (fig. S4D). The cell wall-related DEGs included *CELLULOSE SYNTHASES (CESAs)*, *CELLULOSE SYNTHASE INTERACTIVE1 (CSI1)*, and cell-wall loosening enzymes such as *EXPANSINS* and *XYLOGLUCAN ENDOTRANSGLUCOSYLASES*. Cell-wall-related genes such as *CESAs* have been demonstrated to be direct targets of BES1 and BZR1 (19, 20, 40), but their spatiotemporal regulation, especially in the cortex, has not been reported.

To monitor their responsiveness to brassinosteroids, we generated transcriptional reporters for three of the DEGs with distinct spatiotemporal patterns (Fig. 1C and fig. S5A–E). For example, *CELL WALL / VACUOLAR INHIBITOR OF FRUCTOSIDASE 2 (C/VIF2)* was enriched in the transition domain and elongation zone of the cortex and induced by BL (Fig. 1C). These results confirm that our differential expression analysis captures spatiotemporal brassinosteroid responses and raise the possibility that brassinosteroid induction of cell-wall-related genes is associated with cortex cell elongation.

### Induction of cell wall-related genes is associated with switch to elongation

To better understand how brassinosteroids influence cell wall-related gene expression, we performed scRNA-seq at six time points beginning with BRZ treatment (time 0) and BL treatments for 30 minutes, 1 hour, 2 hours, 4 hours, and 8 hours (Fig. 1, D and E). These time points capture the rapid root elongation triggered by the re-addition of brassinosteroids (35). Waddington-Optimal Transport (WOT) is an analytical approach for analyzing expression trends over a scRNA-seq time course. WOT connects snapshots of gene expression to facilitate trajectory reconstruction, identifying putative ancestors for a given set of cells at earlier time points and descendants at later time points (41).

**Fig. 2.**
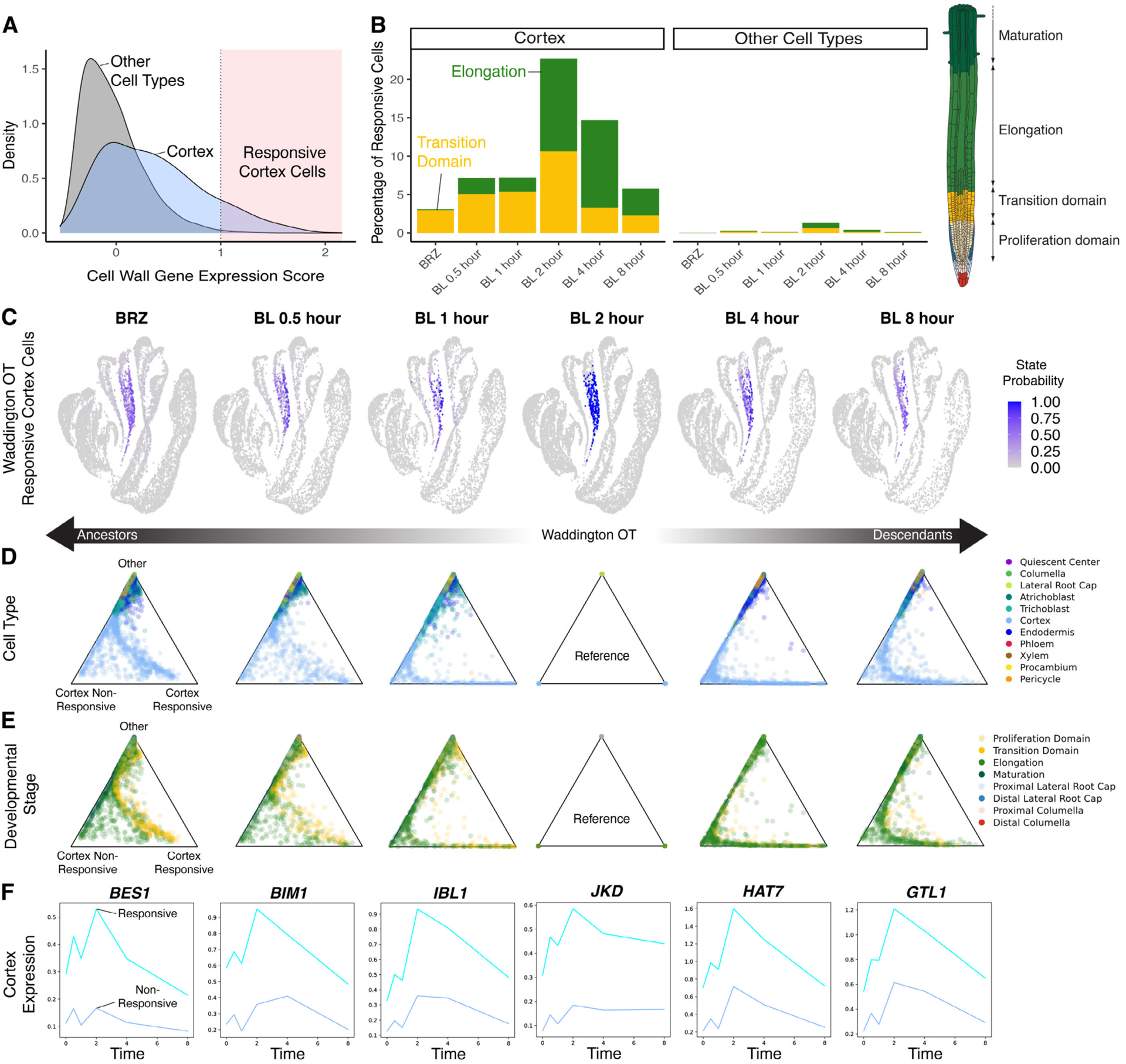
Waddington-OT traces the induction of cell wall-related genes along cortex trajectories associated with the switch to elongation. **(A)** Density plot showing cell wall gene expression score. The shaded region with cell wall expression scores >=1 indicates “responsive cortex cells”. **(B)** Bar plot showing the percentage of responsive cells in the cortex versus other cell types over the time course. Color indicates developmental stage annotation, also depicted in the root schematic. Illustration adapted from the Plant Illustrations repository. Only transition and elongation zones are plotted, as other zones represent less than 2% of responsive cells. **(C)** WaddingtonOT (WOT) probabilities for cortex responsive state along the BL time course. The BL 2-hour time point was used as a reference, therefore all cells have a probability of either 1 or 0 at this time point. **(D-E)** Triangle plots with cells plotted according to WOT cortex responsive, cortex non-responsive, or other state probabilities for each time point along the BL time course. Color indicates the cell type (D) or developmental stage (E) annotation. (F) Expression trends for select transcription factors differentially expressed along WOT cortex responsive trajectories.

To examine the trajectories leading to the activation of cell wall-related genes in the elongating cortex, we applied WOT (41) and created a cell wall gene signature using 107 cell wall-related genes that were induced by BL in the elongating cortex. We monitored the relative expression of these genes, resulting in a “cell wall score” for each cell in the time course (see methods). Cortex cells had a higher cell wall score compared to other cell types, which increased with BL treatment (Fig. 2A–B and fig. S6A–B), confirming that the cell wall score represents a brassinosteroid-responsive module in the cortex. At the 2-hour BL time point, more than 20% of cortex cells had a cell wall score greater than 1. In contrast, only 5% or fewer cells in other cell types exhibited scores this high (Fig. 2B). We therefore designated cells with a cell wall score of at least 1 as “responsive cortex cells” to indicate their exceptional brassinosteroid response (Fig. 2A).

An advantage of WOT analysis is that it does not rely on pre-specified boundaries between developmental zones. We used this property to examine the relationship between developmental stage annotation and cell wall score. Under BRZ treatment, responsive cortex cells were sparse and predominately annotated as transition domain. Upon BL treatment, the annotation of responsive cortex cells shifted to the elongation zone (Fig. 2B). Using the cells at the 2 hour time point as a reference, we looked at the probability of cells being ancestors or descendants of responsive cortex cells (Fig. 2C). We also constructed a similar trajectory for the remaining cortex cells, which were designated “non-responsive cortex cells”. We visualized these probabilities on barycentric coordinates as triangle plots where vertices represent a 100% chance of becoming the labeled state, and interior positions indicate intermediate probabilities. Cells can be colored according to gene expression, cell types, or other quantities. The cortex non-responsive state was represented by the lower left vertex, the cortex responsive state on the lower right vertex, and the top vertex represented other states. This illustrated that cortex transition domain cells were predisposed toward the responsive state under BRZ conditions and subsequently shifted from transition domain to elongation zone annotation upon BL treatment (Fig. 2, D and E). These results suggest that brassinosteroids are involved in initiating the elongation of cortex cells via the activation of cell wall-related genes.

### WOT trajectories identify brassinosteroid-responsive transcription factors

To reveal potential regulators of cell wall-related genes in the cortex, we performed probabilistic differential expression analysis along WOT trajectories, contrasting cells assigned to cortex responsive versus non-responsive states at each time point (see methods). Among the DEGs identified were known transcription factors in the brassinosteroid pathway, including *BES1* (17), *BES1-INTERACTING MYC-LIKE1* (*BIM1)* (42), and *IBH1-LIKE 1* (*IBL1*) (43); Fig. 2F). We also identified additional transcription factors including *JACKDAW* (*JKD*), which is involved in ground tissue specification (44), the class I HD-ZIP transcription factor *HAT7* (45, 46) and *GTL1*. Because *JKD* was identified by WOT and a previous BL RNA-seq dataset (21) but not pseudobulk differential expression analysis, we confirmed its brassinosteroid-responsiveness (fig. S6, C and D). These results indicate that WOT trajectories can identify brassinosteroid-responsive transcription factors that may be involved in regulating cell wall-related genes in the cortex.

### Analysis of the triple receptor mutant *bri1-T* reveals changes in cortex expression

Since our results indicated that exogenous brassinosteroids lead to the activation of cell wall-related genes in the elongating cortex, we asked if this is also the case for endogenous brassinosteroids. A gradient of brassinosteroids is present along the longitudinal axis of the root, with low brassinosteroid levels in the proliferation domain (47). Brassinosteroid biosynthesis increases as cells enter the transition domain and peaks in the elongation zone, shootward of which is a brassinosteroid signaling maximum (35, 47). Interpretation of this endogenous brassinosteroid gradient requires receptor BRI1 and its homologs BRL1 and BRL3 (13, 14, 48–50).

**Fig. 3.**
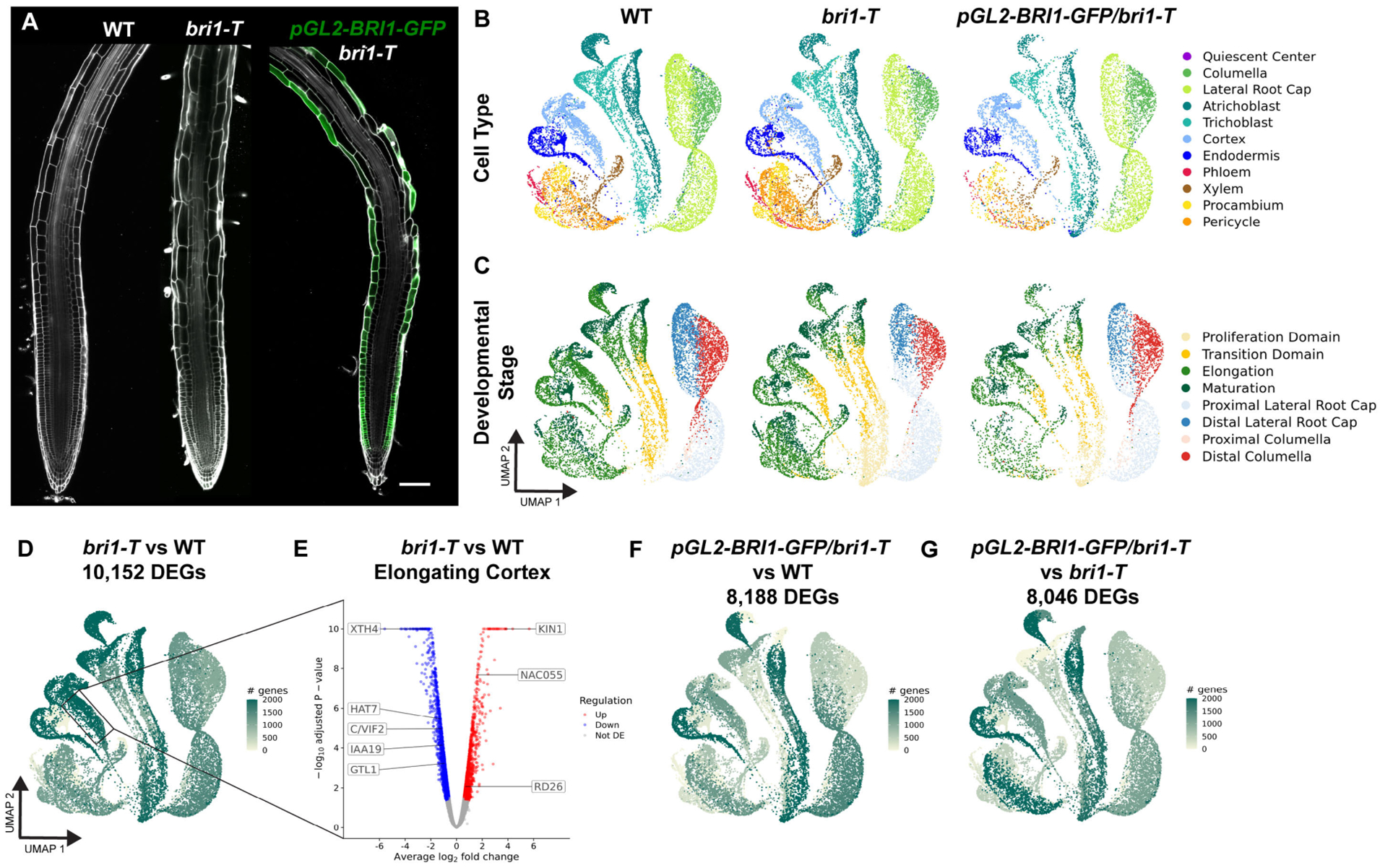
Triple receptor mutant *bri1-T* gene expression changes in cortex and distinct patterns in *pGL2-BRI1-GFP/bri1-T*. **(A)** 7-day old WT, *bri1-T* and *pGL2-BRI1-GFP/bri1-T* roots grown under control conditions. Propidium iodide-staining is shown in grey, and GFP in green. Scale bars, 100 μm. **(B)** UMAP projection of scRNA-seq from 14,334 wild-type cells, 12,649 *bri1-T* cells and 7,878 *pGL2-BRI1-GFP/bri1-T* cells. Two biological replicates of scRNA-seq were performed for each genotype. Colors indicate cell type annotation. **(C)** UMAP projection colored by developmental stage annotation. **(D)** UMAP colored by DEGs for each cell type/developmental stage combination of *bri1-T* compared to WT. **(E)** Volcano plot of DEGs in the elongating cortex from *bri1-T* compared to WT showing down-regulation of cell wall-related genes in *bri1-T*. Color indicates the direction of regulation. **(F-G)** UMAP colored by DEGs for each cell type/developmental stage combination of *pGL2-BRI1-GFP/bri1-T* compared to WT (F) or *pGL2:BRI1-GFP/bri1-T* compared to *bri1-T* (G).

To identify differentially expressed genes, we performed two replicates of scRNA-seq on the brassinosteroid-blind *bri1brl1brl3* triple mutant (*bri1-T)* along with paired wild-type controls (Fig. 3A–C). A previous study profiled single cells from *bri1-T*, suggesting potential brassinosteroid-responsiveness of the cortex (51). However, these data were from a single replicate, were compared to a wild-type sample from a different study, and did not resolve developmental stage-specificity. In contrast, our analysis across both cell types and developmental stages identified the elongating cortex as exhibiting differential gene expression (Fig. 3, D and E, fig. S7A and Data S3). The genes down-regulated in the elongating cortex of *bri1-T* were enriched for the GO term “cell wall organization or biogenesis” (fig. S7B). BL treatment increased the proportion of elongating cortex cells (fig. S7C), but fewer elongating cortex cells were observed in *bri1-T* compared to WT (fig. S7D).

To further verify these changes in gene expression, we performed an additional side-by-side scRNA-seq experiment in which we inhibited endogenous brassinosteroids via BRZ treatment. We profiled 16,642 WT control and 14,320 WT BRZ-treated cells and detected 6,928 DEGs in BRZ vs control using pseudobulk differential expression analysis of each cell type/developmental stage combination (fig. S8A–C). Consistent with the idea that brassinosteroids promote cell wall-related gene expression in the elongating cortex, BRZ down-regulated genes in the elongating cortex were enriched for genes associated with “cell wall organization or biogenesis” (fig. S8D). Together our analysis of *bri1-T* and BRZ treatment indicates that endogenous brassinosteroid signaling promotes the expression of cell wall-related genes in the cortex associated with the onset of elongation.

The epidermis is widely described as the major site for brassinosteroid-promoted gene expression in the root (9, 10, 24, 27, 35, 52). Previous studies showed that epidermal expression of BRI1 was sufficient to rescue morphological phenotypes, including meristem size of *bri1-T* (9, 24, 53). To determine the extent to which brassinosteroid-regulated gene expression is restored, we performed scRNA-seq on *pGL2-BRI1-GFP/bri1-T* - a line in which BRI1 is expressed in atrichoblast cells of the epidermis of *bri1-T* (9, 27, 53). We identified 8,188 DEGs in comparison with wild type (Fig. 3F) and 8,046 DEGs in comparison with *bri1-T* (Fig. 3G and fig. S9A–D), indicating that gene expression remains perturbed (fig. S10A–D) and that this is far from a complete rescue of the *bri1-T* phenotype.

### Tissue-specific CRISPR confirms role of cortex in brassinosteroid-mediated cell expansion

**Fig. 4.**
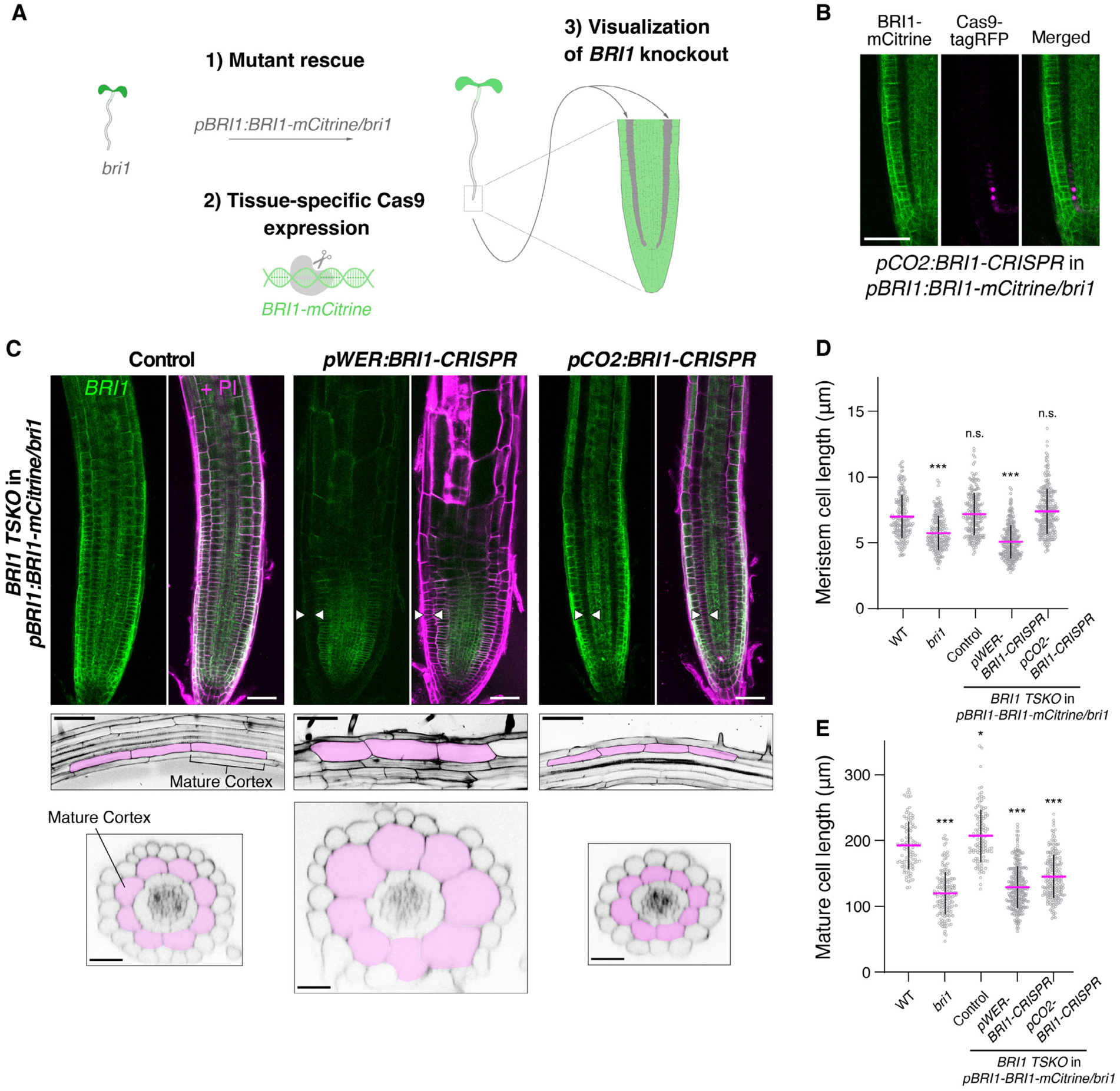
Tissue-specific CRISPR of *BRI1* confirms role for cortex in brassinosteroid-mediated cell expansion. **(A)** Overview of BRI1 tissue-specific CRISPR approach. A *bri1* mutant complemented with *pBRI1-BRI1-mCitrine* (1) was used as background to introduce tissue-specific Cas9 along with gRNAs targeting BRI1 (2). This allows for visualization of BRI1 knockout in specific cell layers, such as the cortex when *pCO2-BRI1-CRISPR* is used (3). **(B)** Appearance of Cas9-tagRFP in the cortex is associated with loss of BRI1-mCitrine signal, confirming tissue-specific knockout. **(C)** Confocal images of BRI1 tissue-specific CRISPR lines. Control indicates a broad expression pattern of BRI1-mCitrine in pBRI1-BRI1-mCitrine/*bri1*. BRI1-mCitrine signals are shown in green, and propidium iodide staining (PI) in magenta (upper panels). White arrows specify tissues with absence of BRI1-mCitrine signal; epidermis for *pWER-BRI1-CRISPR* and cortex for *pCO2-BRI1-CRISPR*. Mature root longitudinal and cross sections illustrate changes in cell length (middle panels) and width (lower panels), respectively. Cortex cells are pseudocolored to indicate their position. **(D)** Quantification of meristematic cortex cell length, defined as the first 20 cells of individual roots starting from the quiescent center. Control indicates pBRI1-BRI1-mCitrine/*bri1* complemented line. **(E)** Quantification of mature cortex cell length. For (D) and (E), all individual data points are plotted. Magenta horizontal bars represent the means, and error bars represent s.d. Significant differences between each line and wild type were determined by one-way ANOVA and Dunnett’s multiple comparison tests. *** P < 0.001, ** P < 0.01 and * P < 0.05. n.s. not significant. Scale bars, (B) and upper panels (C) 50 μm, middle panels (C) 100 μm and lower panels (C) 25 μm. TSKO, tissue-specific knockout.

Characterization of cell-type-specific brassinosteroid signaling has relied on tissue-specific complementation lines, which led to conflicting results and overlooked the role of brassinosteroid signaling in the cortex (9, 24, 27, 35, 51, 53, 54). To selectively block brassinosteroid signaling in cell types of interest, we performed tissue-specific CRISPR (6) of *BRI1*. We used a *bri1* mutant complemented with *pBRI1-BRI1-mCitrine* (Fig. 4A) into which we introduced *Cas9* driven by tissue-specific promoters to knock out *BRI1* either in the epidermis and lateral root cap (*pWER-BRI1-CRISPR*) or in the cortex (*pCO2-BRI1-CRISPR*). mCitrine signals were absent in the expected locations of the tissue-specific CRISPR lines, confirming their efficacy and specificity (Fig. 4A–C and fig. S11A–C).

Since our scRNA-seq data indicated that brassinosteroids promote the expression of cell wall-related genes in the elongating cortex, we hypothesized that loss of brassinosteroid signaling in the cortex would affect final cell size. Indeed, *pCO2-BRI1-CRISPR* lines displayed significantly shorter mature cortex cells, while meristematic cortex cell length was relatively unaffected (Fig. 4D–E).

In contrast, epidermal knockout of BRI1 in *pWER-BRI1-CRISPR* lines resulted in both reduced meristem cell size and reduced mature cortex cell length (Fig. 4D–E), which is consistent with the reported role of epidermal brassinosteroid signaling (9, 25, 27, 35, 52) and brassinosteroid-responsiveness across developmental zones of the epidermis in our scRNA-seq data.

We measured the cell width of the tissue-specific CRISPR lines and found that pWER-BRI1-CRISPR lines have significantly wider cortex cells. However, despite their reduced cortex cell length, *pCO2-BRI1-CRISPR* lines did not have increased cell width (fig. S11D). This prompted us to perform 3D cell segmentations using MorphoGraphX (55, 56). We found that the cortex cell volume was reduced in *pCO2-BRI1-CRISPR* roots but increased in *pWER-BRI1-CRISPR* roots (fig. S12, A and B). Brassinosteroids primarily control cell elongation rather than cell volume when signaling is affected across the entire root(51, 52). On the other hand, the changes in volume observed in the tissue-specific CRISPR lines could be due to constraints between tissues or tissue-specific functions of brassinosteroids in influencing cell volume. These results indicate that in addition to the epidermis, brassinosteroid signaling in the cortex is required to promote cell expansion in the elongation zone. The cortex could instruct anisotropic growth through its physical connection with the epidermis, but as the outermost tissue, relaxation of the epidermis appears to be required to allow for cell elongation (57, 58). This may explain the widening of cortex cells in *pWER-BRI1-CRISPR* lines.

*BRI1* was also reported to rescue *bri1-T* morphology when expressed in the developing phloem using the *CVP2* promoter (51, 53, 54). However, gene expression was not fully restored to wild-type levels in either epidermal or phloem rescue lines. Our scRNA-seq of epidermal *pGL2-BRI1-GFP/bri1-T* lines showed gene expression patterns distinct from either wild type or *bri1-T*. Similarly, scRNA-seq of *pCVP2-BRI1-CITRINE/bri1-T* indicated an intermediate state between wild type and *bri1-T* (51). BRI1 driven by its native promoter was still present in the stele of our tissue-specific CRISPR lines when we observed phenotypic defects, suggesting that, unlike *pCVP2-BRI1*, native expression of *BRI1* in the stele is not sufficient for brassinosteroid-induced cell elongation and root growth. These results confirm the role of the epidermis in brassinosteroid-regulated root growth and reveal the function of the cortex in brassinosteroid-mediated cell elongation, demonstrating how scRNA-seq can identify a spatiotemporal context for hormone signaling.

### *HAT7* and *GTL1* are brassinosteroid-responsive regulators along cortex trajectories

**Fig. 5.**
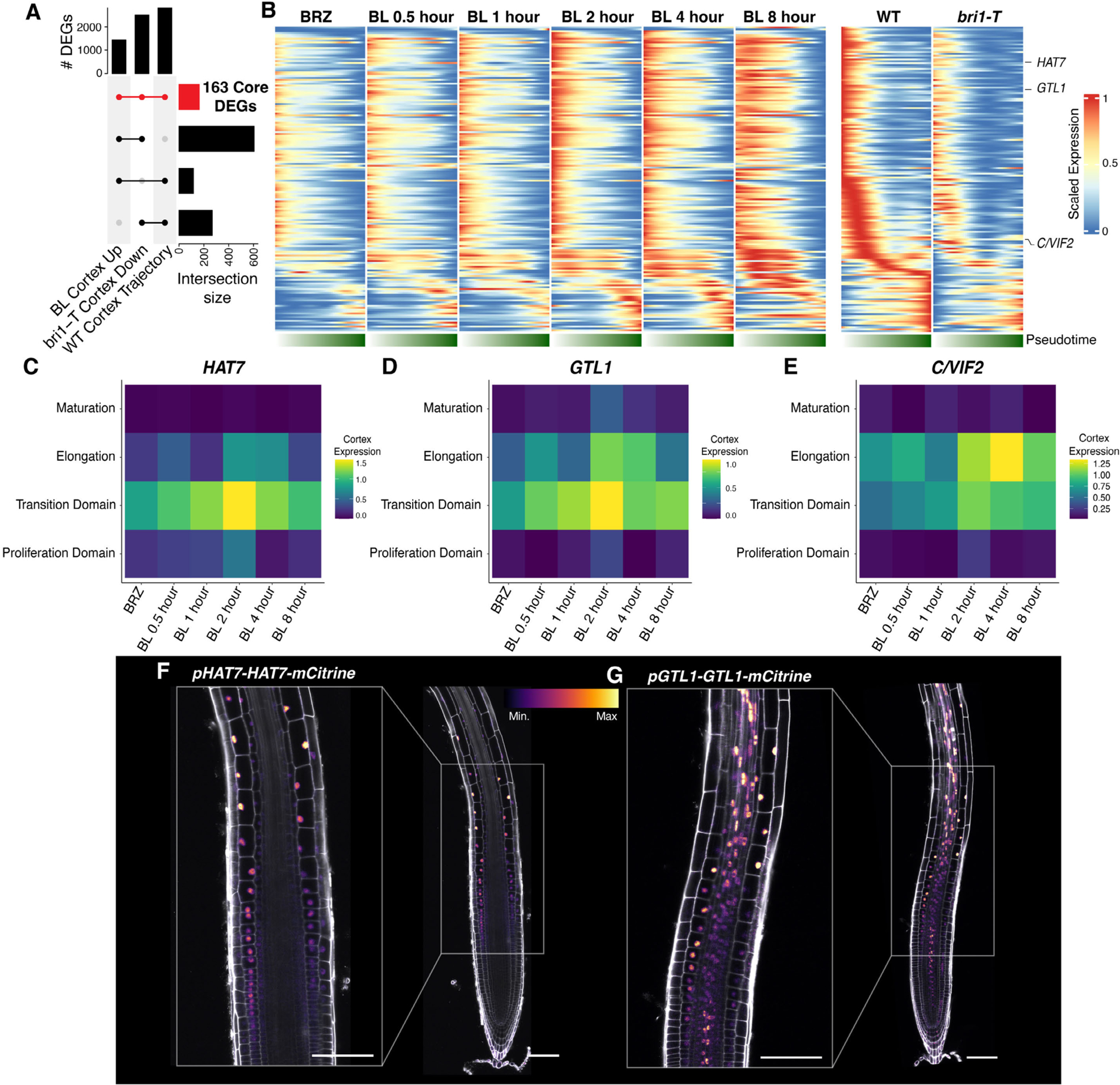
*HAT7* and *GTL1* are brassinosteroid-responsive regulators along cortex trajectories **(A)** Upset plot showing a comparison of genes up-regulated by BL in the cortex, down-regulated in the cortex of *bri1-T*, and differentially expressed along wild-type cortex trajectories. The red color indicates 163 genes common to all three sets. **(B)** Gene expression trends for 163 core brassinosteroid DEGs along cortex trajectories. Scaled expression along cortex pseudotime is plotted for each time point of the brassinosteroid time series and for wild type versus *bri1-T*. Lower bar indicates pseudotime progression calculated by CytoTRACE. **(C-E)** Gene expression trends for *HAT7, GTL1*, or *C/VIF2* along the developmental zones of the cortex for each time point of the brassinosteroid time course. Color bar indicates the scaled expression level in the cortex. **(F-G)** 7-day-old roots expressing *pHAT7-HAT7-mCitrine* (F) or *pGTL1-GTL1-mCitrine* (G) reporters under control conditions show an increased expression as cortex cells elongate. Propidium iodide-staining is shown in grey, with the color gradient indicating relative mCitrine levels. Scale bars, 100 μm.

To define a core set of genes associated with brassinosteroid response along cortex trajectories, we first compared genes induced in the cortex by BL treatment with those down-regulated in the cortex of *bri1-T*. Of the 768 genes in common, we then asked which vary with development in wild-type cortex trajectories (31). The intersection of these three lists identified a core set of 163 brassinosteroid-responsive DEGs (Fig. 5A and Data S3). Consistent with regulation by brassinosteroids, 69% of the core DEGs are BES1 and BZR1 direct targets from ChIP experiments (19, 20, 59). Expression along cortex pseudotime illustrates induction by BL treatment and down-regulation in *bri1-T* (Fig. 5B). *HAT7* and *GTL1* were induced along these trajectories, suggesting a potential role for these transcription factors in controlling brassinosteroid-regulated gene expression in the cortex (Fig. 5C–E).

To gain insight into their roles, we generated translational reporter lines for HAT7 and GTL1. Under control conditions, *pHAT7-HAT7-mCitrine* lines showed expression in the transition domain and elongation zone of the cortex (Fig. 5F). We also observed HAT7 signals in the epidermis and endodermis, in line with expression patterns in our wild-type scRNA-seq atlas (31). *HAT7* expression was decreased when brassinosteroid biosynthesis was inhibited with BRZ and restored upon BL treatment (fig. S13A).

*pGTL1-GTL1-mCitrine* was more broadly expressed, with increasing levels in the cortex as cells progress from the transition domain to the elongation zone (Fig. 5G). GTL1-mCitrine expression was reduced by BRZ and increased by BL treatment (fig. S13B). These results confirm that brassinosteroids promote the expression of *HAT7* and *GTL1*, coinciding with the onset of cell elongation. Furthermore, *HAT7* and *GTL1* are direct targets of BES1 and BZR1 (19, 20, 59), suggesting that they may be part of the brassinosteroid-directed GRN activated as cells progress from proliferation to elongation.

Previous studies inferred global (19, 20, 22, 23) or temporally resolved GRNs (21) for brassinosteroid response, but they lacked cell type and developmental-stage specificity. To infer GRN configurations across our brassinosteroid time series, we used CellOracle (see methods), focusing on BL DEGs and associated transcription factors.

**Fig. 6.**
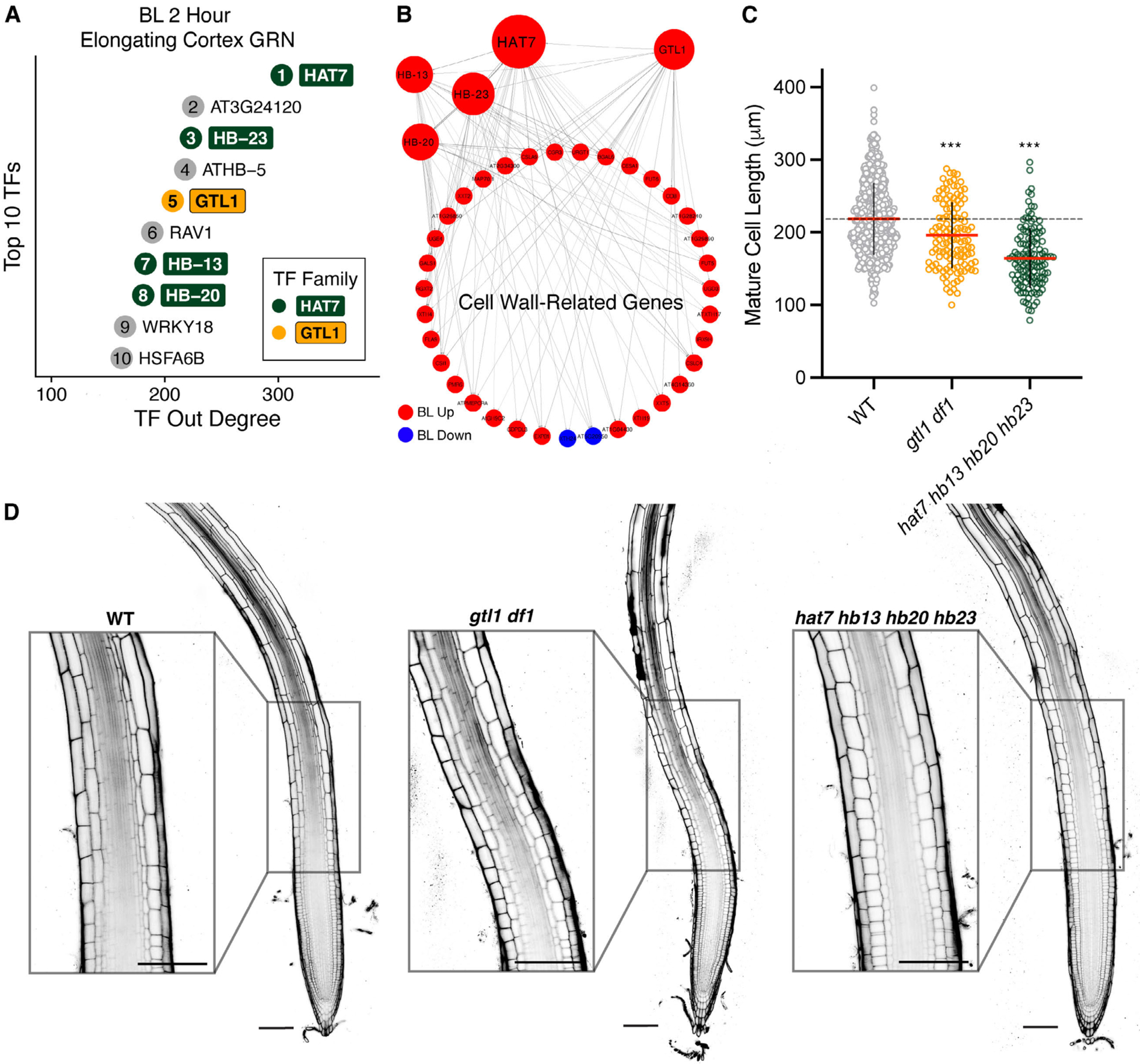
HAT7 and GTL1 are top-ranked regulators in cortex GRNs and affect brassinosteroid-related phenotypes. **(A)** Top 10 transcription factors in the CellOracle BL 2-hour elongating cortex GRN ranked by out-degree. Ranking is indicated by the number inside the circle. Color indicates transcription factor family, with light grey corresponding to any family other than HAT7 or GTL1. **(B)** Subnetwork showing cell wall-related genes that are predicted targets of HAT7 and GTL1 in the CellOracle elongating cortex GRN. HB13, HB20, and HB23 are included in the subnetwork since they are connected to HAT7 and cell-wall-related genes. Node size is proportional to degree. **(C)** Quantification of mature cortex cell length. Red horizontal bars represent the means, and error bars represent s.d. Significant differences between each line and wild type were determined by one-way ANOVA and Dunnett’s multiple comparison tests. ***P< 0.001. **(D)** Propidium iodide-staining of 7-day-old WT, *hat7 hb13 hb20 hb23* (line 1–2), and *gtl1 df1* roots. Insets show cortex cells entering the elongation zone. Scale bars, 100 μm.

Analysis of network importance scores, such as centrality measures, is a powerful approach to prioritize candidate regulators among DEGs (60). Since the cell wall signature peaked at 2 hours after BL treatment, we prioritized transcription factors with high network centrality scores in the elongating cortex at this time point. *HAT7* was the top-ranked transcription factor in terms of degree centrality. *HB13*, *HB20*, and *HB23* were also among the top 10 transcription factors (Fig. 6A–B and Data S4).

We used CRISPR to generate *hat7* loss-of-function mutants but did not observe phenotypes concerning cortex cell elongation (Fig. S14A–C). Together HAT7, HB13, HB20 and HB23 make up the alpha clade HD-ZIP I transcription factors(45, 46, 61–65). Since *HB13*, *HB20* and *HB23* are induced by brassinosteroids and predicted to regulate cell wall-related genes in our GRNs (Fig. 6B, fig. S15A–B and Data S5), we next generated *hat7 hb13 hb20 hb23* quadruple mutants via multiplex CRISPR. Mature cortex cell length was reduced by approximately 25% in two independent quadruple mutants (Fig. 6C–D and fig. S16A–C), providing evidence that HAT7 and its homologs are required for cell elongation. *hat7 hb13 hb20 hb23* quadruple mutants also had wider mature cortex cells (fig. S16C) with increased volume compared to wild-type (fig. S17, A and B). Despite the decrease in final cell length, the root length of the quadruple mutant was not reduced (fig. S14A), suggesting that the decrease in cell length is at least partially compensated for by increased cell production. We found that *hat7 hb13 hb20 hb23* quadruple mutants have an average of 15 more meristematic cortex cells compared to wild-type (fig. S16B), which is consistent with a compensatory increase in proliferation.

We next investigated GTL1, the 5th highest-ranked transcription factor in the BL 2-hour elongating cortex GRN (Fig. 6A–B). Given that GTL1 was shown to function redundantly with DF1 in terminating root hair growth (66, 67), we examined *gtl1 df1* double mutants, finding shorter mature cortex cell lengths and shorter roots compared to wild-type (Fig. 6C–D and fig. S14A–C). *DF1* was challenging to detect in scRNA-seq (fig. S15A) due to its low expression level (66). However, we observed increasing trends of *DF1* expression along WOT trajectories in the BL time course, especially in cell wall responsive cortex cells (fig. S15B), which was verified using a p*DF1-DF1-GFP* reporter (fig. S15C).

We examined the overlap between our CellOracle BL 2-hour elongating cortex GRN and a GRN reported by Clark et al. from bulk RNA-seq data in response to brassinosteroids (21). Only 43/31,330 edges are shared between the two networks. Moreover, HAT7, HB13, HB20, HB23, and GTL1 were among the top 10 transcription factors in terms of out-degree in the CellOracle elongating cortex GRN, which we validated through mutant analysis. On the other hand, none of these were among the top 100 transcription factors in the Clark et al. GRN (fig. S18, A and B), suggesting that they would have been difficult to prioritize from the bulk data alone. Together, our genetic analysis of HAT7 and GTL1 family transcription factors illustrates the power of GRN-mediated discovery of regulatory factors in spatiotemporal brassinosteroid response.

### BES1 and GTL1 physically interact and regulate shared target genes

Since BES1 is known to interface with other transcription factors in controlling brassinosteroid-regulated gene expression, we compared target genes for BES1 and BZR1 (19, 20, 59) to ChIP targets of GTL1 and DF1 (66). BES1 and BZR1 share 3,020 common targets with GTL1 and 2,490 common targets with DF1 (fig. S19A). When compared to brassinosteroid-regulated genes from scRNA-seq, BES1 and GTL1 targets showed the strongest enrichment in genes up-regulated by brassinosteroids in the transition domain and elongation zone of the cortex (fig. S19B), with 297 common targets of both BES1 or BZR1 and GTL1 being induced in the elongating cortex by BL treatment.

Given the overlap between BES1 and GTL1 targets, we hypothesized that these transcription factors physically interact to regulate a common set of genes. Co-immunoprecipitation showed that GTL1-FLAG pulled down BES1-GFP (fig. S19C). These results suggest that brassinosteroids induce GTL1 and subsequently BES1 and GTL1 interact to control a common set of target genes. This type of feed-forward loop could provide a mechanism to amplify the brassinosteroid signal and/or to direct BES1 to drive tissue-specific gene expression by interacting with other more specifically expressed transcription factors.

In addition to *GTL1*, we found that previously described BES1 interacting transcription factors including *BRASSINOSTEROIDS AT VASCULAR AND ORGANIZING CENTER* (*BRAVO*) (26), *BIM1*(42), and *MYB30*(68), were differentially expressed in our scRNA-seq datasets in response to BL (Data S3). Among these, *MYB30* was enriched in atrichoblasts of the epidermis and induced by BL treatment (fig. S20, A and B). CellOracle correctly predicted that *MYB30* regulates *LTPG2*, a known target of *MYB30* (Mabuchi et al., 2018), which also showed enrichment in atrichoblasts and temporal induction in response to brassinosteroids following *MYB30* activation (fig. S20B). This supports the idea that cell type-specific patterns of brassinosteroid response may be at least partially explained by interactions between BES1 or BZR1 and other transcription factors.

### scRNA-seq reveals cell-type-specific expression underlying *gtl1 df1* phenotypes

**Fig. 7.**
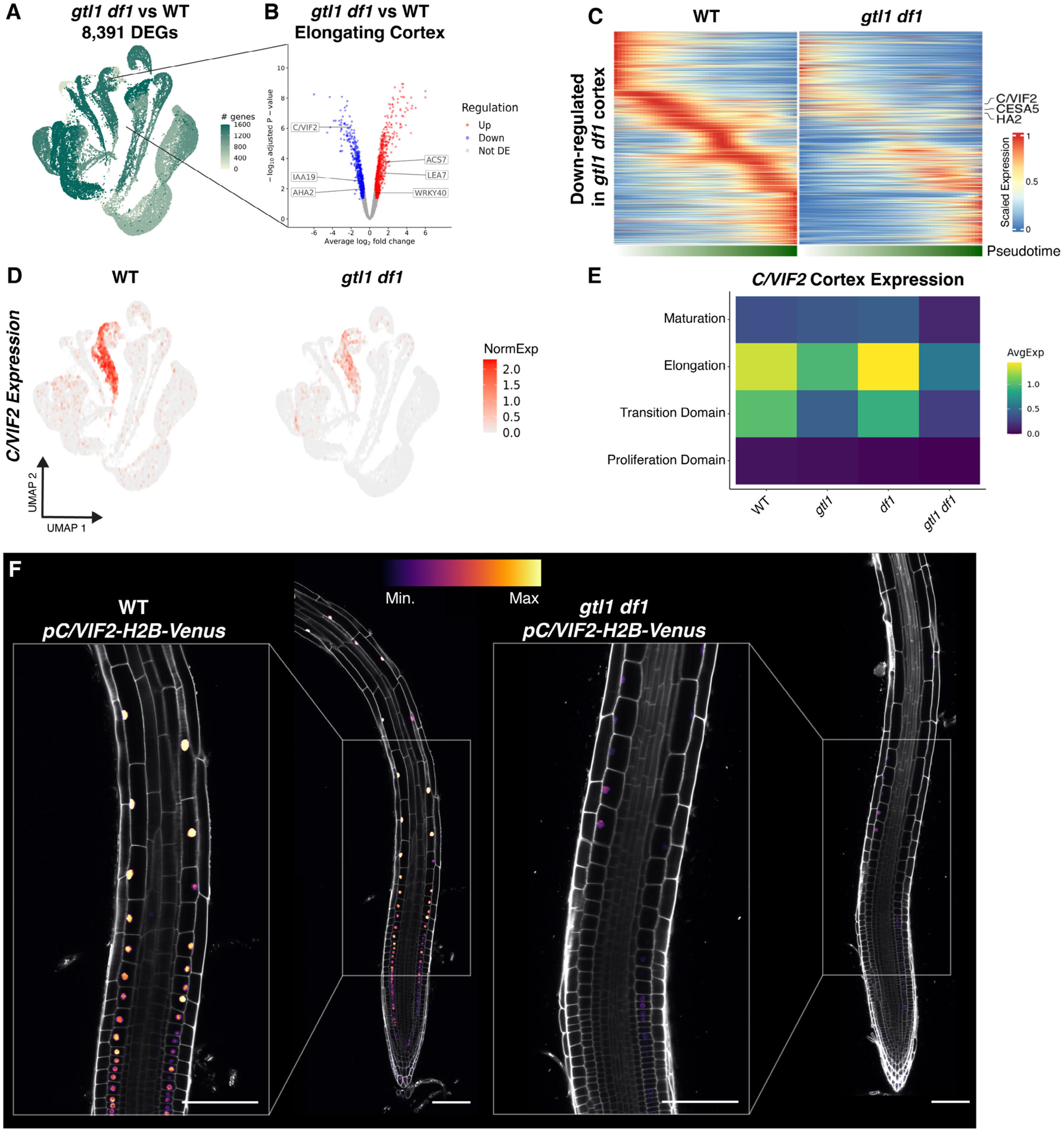
scRNA-seq reveals cell-type-specific expression underlying *gtl1 df1* phenotypes **(A)** UMAP projection of scRNA-seq from 74,810 WT, *gtl1*, *df1*, and *gtl1 df1* cells. Two biological replicates were profiled for each genotype. Color indicates DEGs for each cell type/developmental stage combination of *gtl1 df1* compared to WT. **(B)** Volcano plot of DEGs in the elongating cortex from *gtl1 df1* compared to WT. Color indicates the direction of regulation. **(C)** Gene expression trends along cortex trajectories for down-regulated DEGs in *gtl1 df1* compared to WT. Each row represents the scaled expression of a gene along cortex pseudotime. The lower bar indicates pseudotime progression calculated by CytoTRACE. **(D)** Expression of *C/VIF2* in wild type and *gtl1 df1* scRNA-seq. The color scale represents log normalized, corrected UMI counts. **(E)** *C/VIF2* expression levels plotted along the developmental zones of the cortex for WT, *gtl1*, *df1*, and *gtl1 df1*. The color bar indicates the scaled expression level. **(F)** 7-day-old root images of a *pC/VIF2-H2B-Venus* reporter in wild type or *gtl1 df1* under control conditions. Propidium iodide-staining is shown in grey, with the color gradient indicating relative mCitrine levels. Scale bars, 100 μm.

Our results indicate that *gtl1 df1* mutants have reduced cortex cell elongation. On the other hand, *gtl1 df1* mutants have longer trichoblasts (66). A downstream regulatory network that enables GTL1-mediated growth inhibition has been dissected in trichoblasts (66, 67). To identify the cell-type-specific changes in gene expression underlying *gtl1 df1* cortex phenotypes, we performed scRNA-seq on *gtl1* and *df1* single mutants and the *gtl1 df1* double mutant. Using pseudobulk differential expression analysis, we detected relatively subtle changes in *gtl1* or *df1* single mutants compared to the wild type (fig. S21A–C). In contrast, 8,391 genes were differentially expressed in *gtl1 df1* double mutants versus wild type (Fig. 7A).

1,077 genes were up-regulated across all developmental stages of the cortex of *gtl1 df1*, and 947 genes were down-regulated. The majority of cortex DEGs were affected in the elongation zone (Fig. 7, A and B, fig. S21C and Data S3). Of the down-regulated genes in the cortex of the double mutant, 226 genes were also up-regulated by BL treatment. Furthermore, 31.3% of the core brassinosteroid DEGs were down-regulated in the cortex of *gtl1 df1*, whereas only 6.8% were up-regulated (Data S3). These results suggest that GTL1 and DF1 promote the expression of a subset of brassinosteroid-induced genes in the cortex.

Plotting *gtl1 df1* DEGs along cortex pseudotime illustrated the down-regulation of several genes involved in cell elongation including *CESA5* and *AHA2* (Fig. 7C). These genes were enriched for the GO term “cell wall organization or biogenesis” (fig. S21D). We next examined *C/VIF2*, because it is induced by BL in the cortex, but its expression decreased in cortex cells of *gtl1 df1* (Fig. 7, D and E). A *pC/VIF2-H2B-Venus* reporter showed expression of *C/VIF2* in the transition and elongation zone of the wild-type cortex, whereas its expression was reduced in the cortex of *gtl1 df1* mutants (Fig. 7F and Movie S1). The reduced expression of cell wall-related genes in *gtl1 df1* mutants validates our cell-type-specific brassinosteroid GRNs and identifies a function of GTL1 in promoting cortex cell elongation in response to brassinosteroids.

## Discussion:

Understanding how hormone-mediated GRNs are controlled in space and time has the potential to enable the engineering of specific downstream responses to optimize plant growth under a changing environment (10, 69). Plant hormones including brassinosteroids, auxin, gibberellins, and abscisic acid have been shown to exhibit tissue-specific responses (70–75), but how the associated GRNs are modulated in different cell types at particular developmental stages is enigmatic. In this study, we profiled brassinosteroid responses across cell types, developmental stages, and time points of treatment using scRNA-seq, providing a high-resolution map of signaling outputs. These data are publicly available as an interactive browser (https://shiny.mdc-berlin.de/ARVEX/). We identified the elongating cortex as a spatiotemporal context for brassinosteroid signaling, where brassinosteroids activate cell wall-related genes and promote elongation. We further showed that HAT7 and GTL1 are brassinosteroid-induced regulators along cortex trajectories that control cell elongation. These findings highlight the ability of single-cell genomics to identify context-specific transcription factors, a capability that could be leveraged to precisely engineer plant growth, development, and responses to stress. Our results reveal spatiotemporal brassinosteroid responses and the underlying GRNs.

## Methods Summary

*Arabidopsis* accession Columbia-0 (Col-0) was used as a wild type. The following lines were previously described: *bri1 GABI_134E10* (76); *bri1-116brl1brl3* triple mutant (*bri1-T*) (50); *pGL2-BRI1-GFP/bri1-T* (27); *gtl1-1* (WiscDsLox413-416C9), *df1-1* (SALK_106258), and *gtl1-1 df1-1 *(66); JKD-Ypet recombineering line (44). We produced *hat7* single mutants and *hat7 hb13 hb20 hb23* quadruple mutants using FASTRED multiplex CRISPR constructs containing an intronized version of Cas9 (77, 78).

scRNA-seq experiments were performed as previously described (31). For each sample, ~0.5cm root tips from 7-day-old plants were harvested, and digested with protoplasting solution, and 16,000 cells were loaded on a 10X Genomics Chromium instrument, with the aim to capture 10,000 cells per sample with the 10X Genomics Chromium 3` Gene expression v3 or v3.1 kits.

For BL scRNA-seq, we first grew plants on 1 μM BRZ to deplete endogenous brassinosteroids, then transferred plants to either a fresh BRZ plate or 100 nM BL. We performed two separate BL scRNA-seq treatment experiments. The first pilot experiment consisted of a BRZ and 2 hour BL treatment. The second experiment included two additional BRZ and BL 2 hours replicates and a single replicate of the other time points in our time course (BL 0.5, 1, 4, and 8 hour treatments). Each of the BL treatments was staggered so that all samples were collected simultaneously. A total of 70,223 cells were recovered from the BL treatment scRNA-seq experiments. Wild type, *bri1-T*, and *pGL2-BRI1-GFP/bri1-T* were similarly profiled in a side-by-side scRNA-seq experiment under control conditions with two replicates per genotype, resulting in 34,861 cells. To test the effect of inhibiting endogenous brassinosteroid biosynthesis, we grew wild type on 1 μM BRZ or a mock DMSO control and performed two replicates of scRNA-seq spanning 30,962 cells for BRZ vs control analysis. Lastly, scRNA-seq was performed on wild type, *gtl1, df1*, and *gtl1 df1* in duplicate under control conditions resulting in 74,810 scRNA-seq expression profiles.

scRNA-seq data were aligned against the *Arabidopsis* TAIR10 reference genome. Quality filtering of cells was performed using the R package COPILOT (Cell preprOcessing PIpeline kaLlistO busTools) (31, 79). Downstream analysis were carried out using Seurat version 3.1.5 (80), Waddington-Optimal Transport (41), muscat (81), tradeSeq (82), and GRNs inferred using CellOracle (60).

To selectively block brassinosteroid signaling in cell types of interest, we performed tissue-specific CRISPR (6) of *BRI1* in a *bri1* mutant complemented with *pBRI1-BRI1-mCitrine*. Two gRNAs targeting *BRI1* were simultaneously expressed along with tissue-specific Cas9 to knock out *BRI1* either in the epidermis and lateral root cap (*pWER-BRI1-CRISPR*) or in the cortex (*pCO2-BRI1-CRISPR*). For each root used for quantitative analysis, BRI1-mCitrine signal was acquired to confirm the efficiency of the tissue-specific knockout system. The supplementary materials provide all the methods details, including those summarized above.

## Supplementary Material

Supplementary Materials and Methods

Data_S1

Data_S2

Data_S3

Data_S4

Data_S5

Data_S6

Movie_S1

## Data Availability

Single-cell RNA-seq data have been deposited at GEO: GSE212230 and can be visualized through an interactive browser at https://shiny.mdc-berlin.de/ARVEX/. All data are available in the manuscript, the supplementary material or deposited at Zenodo (83, 84).
