# Supplementary material for: Brassinosteroid gene regulatory networks at cellular resolution in the Arabidopsis root
Source: Science. Author manuscript; Available in PMC 2023 Apr 21. (PMC10119888; doi:10.1126/science.adf4721)
Supplement: Supplementary Materials and Methods [file NIHMS1889089-supplement-Supplementary_Materials_and_Methods.pdf]

Science
AAAS

Supplementary Materials for

Brassinosteroid gene regulatory networks at cellular resolution in the *Arabidopsis* root

Trevor M Nolan*, Nemanja Vukašinović*, Che-Wei Hsu*, Jingyuan Zhang, Isabelle Vanhoutte, Rachel Shahan, Isaiah W Taylor, Laura Greenstreet, Matthieu Heitz, Anton Afanassiev, Ping Wang, Pablo Szekely, Aiden Brosnan, Yanhai Yin, Geoffrey Schiebinger, Uwe Ohler, Eugenia Russinova┼, Philip N Benfey┼

*These authors contributed equally to this work.
┼ Corresponding author: Eugenia.Russinova@psb.vib-ugent.be and Philip.Benfey@duke.edu

This PDF file includes:

Materials and Methods
Figs. S1 to S21
Captions for Movie S1
Captions for Data S1 to S6

Other Supplementary Materials for this manuscript include the following:

Movie S1
Data S1 to S6

Materials and Methods

Plant materials and growth conditions

Arabidopsis accession Columbia-0 (Col-0) was used as a wild type. The following lines were previously described: *bri1 GABI_134E10 (76); bri1-116brl1brl3* triple mutant (*bri1-T) (50); pGL2-BRI1-GFP/bri1-T (27); gtl1-1* (WiscDsLox413-416C9), *df1-1* (SALK_106258), and *gtl1-1 df1-1 (66)*; JKD-Ypet recombineering line (*44*). Seeds were sterilized using 50% (v/v) bleach with 0.05% Tween-20 for 10-15 minutes, plated on 1/2 Linsmaier and Skoog (LSP03-1LT, Caisson Labs; pH 5.7), 1% sucrose media, and stratified 2-4 days at 4°C in the dark. Plates were kept vertically in a Percival growth chamber set to 22°C, 16 hours light/8 hours dark, and grown for 7 days unless otherwise indicated. Chemical treatments were conducted by cooling the growth media to approximately 60°C after autoclaving and adding DMSO (a mock solvent), 1 µM Brassinazole (BRZ, SML1406, Sigma) or 100nM Brassinolide (BL, 21594, Cayman Chemical).

bri1-T was maintained as a heterozygote for *bri1-116*, and homozygous mutants were confirmed as previously described (*53*). Primers listed in Data S6 were used to amplify genomic DNA, and the resulting 552bp amplicon was digested with *PmeI*. The mutant *bri1-116* allele could not be digested, whereas WT was cut into 314bp and 238bp fragments.

Transgenic reporters

To generate new reporters for brassinosteroid-responsive genes, we first added the FASTRED seed coat selection cassette (*77*, *85*) and a MoClo (*86*) Level 1 acceptor site to the binary vector pICH86966 (Addgene plasmid #48075). *pHAT7-HAT7-mCitrine* and *pGTL1-GTL1-mCitrine* were assembled into this FASTRED destination vector using Level 1 BsaI golden gate assembly. To facilitate one-step promoter-reporter construction, we assembled an AarI flanked RFP dropout using the overhangs described in the Mobius (*87*) upstream of Venus-H2B followed by the Ubiquitin10 terminator (tUBQ10), a plasma membrane marker (pUBQ10-mScarlet-LTI6-tNos), and a constitutive histone maker (pUBQ10-H2B-CFP-t19s). Promoters containing up to ~3kb of sequence upstream of the ATG start codon for the gene of interest were PCR amplified with AarI containing primers and used to replace the AarI-RFP module in golden gate reactions to generate Promoter-Venus-H2B constructs. Our Venus-H2B reporter included a Ubiquitin tag to decrease reporter perdurance. Although the plasma membrane marker and histone marker were included as positive controls in the constructs, they were not further analyzed in this study. Assemblies were confirmed by restriction digestion and sequencing, transformed into *Agrobacterium*, and used to transform *Arabidopsis* via floral dip (*88*). FASTRED positive T1 seeds were selected under a fluorescent dissecting scope, and only lines with 3:1 segregation of seed coat fluorescence in the T2 generation were used. T2 lines with bright seed fluorescence were typically homozygous in our conditions. Therefore, we used bright T2 seeds or homozygous T3 seeds for experiments. We ensured that reporter signals were consistent across at least three independent transgenic lines.

Generation of mutant lines using multiplex CRISPR

We produced *hat7* single mutants and *hat7 hb13 hb20 hb23* quadruple mutants using FASTRED multiplex CRISPR constructs containing an intronized version of Cas9 (*77*, *78*). Two gRNAs were designed per gene using CHOP-CHOP (*89*). gRNA-containing oligos were hybridized and cloned into pDGE sgRNA shuttle vectors using *BpiI* (Data S6). Each of the gRNA-containing

shuttle vectors were then assembled into pDGE666 (Addgene plasmid # 153231) using BsaI golden gate assembly, sequence verified, and transformed into wild-type *Arabidopsis* as described above. We selected FASTRED positive T1 seeds and subsequently screened FASTRED negative (putatively Cas9-free) T2 seeds for frameshift mutations using Sanger sequencing coupled with ICE analysis of CRISPR edits (*90*). The edits were similarly confirmed in the T3 generation, and at least two homozygous alleles from independent lines were used for experiments.

BRI1 tissue-specific CRISPR

Two gRNAs targeting BRI1 were simultaneously expressed along with tissue-specific Cas9 (*6*). Primers used for cloning of gRNA BRI1-2 (*91*) and gRNA BRI1-3 can be found in Data S6. The entry module pGG-B-AtU6-26-BRI1-2-C and pGG-A-AtU6-26-BRI1-3-B were generated by annealing oligos for each gRNA and ligating into *BbsI*-digested (New England Biolabs) Golden Gate entry vectors described in (*92*). Next, gRNA modules were combined with pGG-C-linker-G plasmid and cloned into pEN-R2-A-G-L3 by restriction-ligation using BsaI enzyme (New England Biolabs) to obtain pEN-R2- gRNA_BRI1-3-gRNA_BRI1-2-L3. This plasmid was combined with pDONR-L1-Cas9p-tagRFP-L2 (*7*), pDONRL4-L1r carrying either WER or CO2 promoters (*93*), and a destination vector pK8m34GW-FAST (*94*) in a MultiSite Gateway LR reaction (Thermo Fisher Scientific) to obtain expression clones. Expression clones were introduced into the Agrobacterium C58 strain and used to transform *pBRI1-BRI1-mCitrine/bri1* plants (*76*) by floral dip. T2 generation seeds were selected based on the presence of GFP signal in the seed coat, and 7-day-old seedlings were used for phenotypic analysis. For each root used for quantitative analysis, BRI1-mCitrine signal was acquired to confirm the efficiency of the tissue-specific knockout system. Statistical analyses were conducted in GraphPad Prism v.9 software.

Confocal microscopy

Confocal imaging for the majority of experiments was performed using a Zeiss 880 equipped with a 40X objective. Excitation and detection were set as follows: Venus and mCitrine, excitation at 488 nm and detection at 499-571 nm; GFP, excitation at 488 nm and detection at 493–558 nm; PI staining, excitation at 561 nm and detection at 605–695 nm. Confocal images were processed using the Fiji package of ImageJ (*95*). Tile scans were stitched and representative median longitudinal sections for each image are shown. Identical settings were used for images that were directly compared.

For BRI1 TSKO confocal, roots were imaged between a block of agar and cover glass in imaging chambers. Image acquisition was performed with a FluoView1000 inverted confocal microscope (Olympus) equipped with a dry 20X objective (NA 0.75) using 514 nm laser excitation and a spectral detection bandwidth of 500–530 nm for mCitrine and 535 nm laser excitation together with a spectral detection bandwidth of 570– 670 nm for PI. For cell segmentation experiments to assess cortical cell volumes, 7-day-old roots of *pBRI1-BRI1-mCitrine/bri1*, *pWER-BRI1-CRISPR*, and *pCO2-BRI1-CRISPR* transgenic lines were stained using mPS-PI staining protocol (*96*). Five roots were analyzed for each genotype. Samples were imaged using Leica SP8X confocal microscope with a x 25 objective. Mature parts of the roots, as indicated by the presence of root hairs, were imaged, and the distance between the Z scans was set to 0.5 µm. Acquired images were processed using Fiji. Next, images were segmented in MorphoGraphX 2.0 (*55*, *56*) using the Insight Toolkit (https://itk.org) morphological watershed

processes, and 3D meshes of each root were created using the marching cubes 3D workflow. Cortical cells were selected, and all other cell types and segments were manually discarded. Cortex cell volumes of wild-type, *gtl1 df1*, and *hat7hb13hb20hb23* were similarly analyzed with four roots per genotype, except that *pUBQ10-mScarlet-LTI6* signals were used to visualize the cell boundaries using a Zeiss 880 equipped with a 40X objective. mScarlet excitation was at 561 nm, and detection was at 590–660 nm.

For the confocal time-lapse video, 7-day-old wild type and *gtl1 df1* seedlings expressing *pC/VIF2-H2B-VENUS* were placed on ½ MS agar blocks containing PI, placed side-by-side in chambered coverglass (Nunc Lab-Tek, ThermoFisher) and imaged under a vertical ZEISS LSM900 microscope equipped with a Plan-Apochromat M27 20x/ 0.8 n.a. objective. The root tip was imaged every 12 min and automatically tracked with the TipTracker software (*97*). The excitation/emission wavelengths were 514 nm/530-600 nm for Venus and 535 nm/580-650nm for PI.

<u>BES1 and GTL1 Co-Immunoprecipitation (Co-IP)</u>

Co-IP experiments were conducted as previously described (*98*). p35S-FLAG-GTL1 and a p35S-FLAG-GUS negative control were cloned into pGWB412 (*99*) using gateway LR reactions. The following construct combinations were co-transformed into *Arabidopsis* mesophyll protoplasts- p35S-BES1-GFP + p35S-FLAG-GUS; p35S-FLAG-GTL1 + p35S-GFP-GUS; p35S-BES1-GFP + p35S-FLAG-GTL1. After overnight incubation, transformed protoplasts were harvested and homogenized in Co-IP buffer (50 mM Tris– HCl, pH 7.5, 150 mM NaCl, 10% (v/v) glycerol, 0.1% (v/v) Nonidet P-40, 1 mM phenylmethysulfonyl fluoride, 20 mM MG132, and proteinase inhibitor cocktail) for 1 h at 4 °C with rotation. 5 µg FLAG M2 antibody (F1804, Sigma) was pre-bound to 40 µL protein G Dynabeads (10003D, Thermo Fisher Scientific) for 30 min in phosphate-buffered saline (PBS) buffer with 0.02% Tween 20 at room temperature. The beads were washed once with the same PBS buffer and resuspended in Co-IP buffer. After protein extraction, 10 µL of anti-FLAG pre-bound Dynabeads was added to each sample for another 1.5 h incubation at 4 °C with rotation. Dynabeads were precipitated using a DynaMagnetic rack (12321D, Thermo Fisher Scientific) and washed twice with Co-IP buffer with Nonidet P-40 and three times with Co-IP buffer without Nonidet P-40. The IP products were eluted in 2XSDS sample buffer and used for immunoblotting with rabbit anti-GFP (A11122, Invitrogen) and rabbit anti-FLAG antibody (F7425, Sigma–Aldrich) at 1:1,000 dilution.

<u>scRNA-seq profiling of *Arabidopsis* root protoplasts using the 10X Genomics chromium system</u>

scRNA-seq experiments were performed as previously described (*31*) with minor modifications. Plants were grown for 7 days as described above with the addition of 100 µm nylon mesh (Nitex 03-100/44) on the plates to facilitate root collection. For each sample, ~0.5cm root tips were harvested from 1000-3000 roots and placed into a 35mm petri dish containing a 70 µm cell strainer and 4.5mL enzyme solution (1.5% [w/v] cellulase [ONOZUKA R-10, GoldBio], 0.1% Pectolyase [Sigma P3026], 0.4 M mannitol, 20 mM MES (pH 5.7), 20 mM KCl, 10 mM CaCl2, 0.1% bovine serum albumin, and 0.000194% (v/v) beta-mercaptoethanol). The digestion was incubated on an 85 rpm shaker at 25°C for one hour with additional stirring every 15-20 minutes. The resulting cell solution was filtered twice through 40 µm cell strainers and centrifuged for 5 minutes at 500g in a swinging bucket rotor. The pellet was washed with 2mL washing solution (0.4 M mannitol, 20 mM MES (pH 5.7), 20 mM KCl, 10 mM CaCl2, 0.1% bovine serum albumin, and 0.000194% (v/v) beta-mercaptoethanol), centrifuged again at 500g for 3 minutes,

and the pellet resuspended in washing solution at a concentration of ~2000 cells/uL. We loaded 16,000 cells, with the aim to capture 10,000 cells per sample with the 10X Genomics Chromium 3` Gene expression v3 or v3.1 kits. Cell barcoding and library construction were performed following the manufacturer's instructions. cDNA and final library quality were verified using a Bioanalyzer High Sensitivity DNA Chip (Agilent) and sequenced on an Illumina NextSeq 500 or NovaSeq 6000 instrument to produce 100bp paired-end reads.
For BL scRNA-seq, we first grew plants on 1 μM BRZ to deplete endogenous brassinosteroids, then transferred plants to either a fresh BRZ plate or 100nM BL. We monitored the efficacy of these treatments using a constitutively expressed 35S-BES1-GFP line. In agreement with previous reports (*17*, *100*, *101*), BES1-GFP was predominantly present in the cytoplasm under low brassinosteroid conditions resulting from BRZ treatment but accumulated in the nucleus following BL treatment (Fig. S1). We performed two separate BL scRNA-seq treatment experiments. The first pilot experiment consisted of a BRZ and 2-hour BL treatment. The second experiment included two additional replicates of BRZ and BL 2 hours along with a single replicate of the other time points in our time course (BL 0.5, 1, 4, and 8 hour treatments). Each of the BL treatments was staggered so that all samples were collected simultaneously. A total of 70,223 cells were recovered from the BL treatment scRNA-seq experiments.
Wild type Col-0, *bri1-T*, and *pGL2-BRI1-GFP/bri1-T* were similarly profiled in a side-by-side scRNA-seq experiment under control conditions with two replicates per genotype, resulting in 34,861 cells. To test the effect of inhibiting endogenous brassinosteroid biosynthesis, we grew wild-type Col-0 on 1 μM BRZ or a mock DMSO control and performed two replicates of scRNA-seq spanning 30,962 cells for BRZ vs control analysis. Lastly, scRNA-seq was performed on Wild type Col-0, *gtl1, df1*, and *gtl1 df1* in duplicate under control conditions resulting in 74,810 scRNA-seq expression profiles.

scRNA-seq data pre-processing

Raw sequencing reads were demultiplexed from Illumina BCL files to produce FASTQ files for each sample using CellRanger mkfastq (v3.1.0, 10X Genomics). Reads were then aligned against the *Arabidopsis* TAIR10 reference genome to generate a gene-by-cell matrix using the scKB script (https://github.com/ohlerlab/scKB), which incorporates kallisto (*102*) and bustools (*103*, *104*). Quality filtering of cells was performed using the R package COPILOT (Cell preprOcessing PIpeline kaLlistO busTools) (*31*, *79*). COPILOT uses a non-arbitrary scheme to remove empty droplets and dying or low-quality cells. We used one iteration of COPILOT filtering, which adequately separated high-quality cells from the background in our samples based on an examination of barcode rank plots. To address issues with doublets and outliers, the resulting high-quality cells were further filtered to remove the top 1% of cells in terms of UMI counts, and putative doublets were removed with DoubletFinder(*105*) using the estimated doublet rate from the 10X Genomics Chromium Single Cell 3' Reagent Kit user guide.

Normalization, annotation, and integration of scRNA-seq datasets

Downstream analyses were carried out using Seurat version 3.1.5. Samples were first individually processed and examined. Data were normalized using SCTransform (*106*) ,and all detected genes were subsequently retained for analysis, except those from mitochondria, chloroplasts, or those affected by protoplasting (absolute log2 fold-change >= 2) (*31*, *34*). Principal component analysis (PCA) was performed by calculating 50 principal components using the RunPCA function (with approx=FALSE). UMAP non-linear dimensionality reduction

was next calculated via the RunUMAP function using all 50 principal components with parameters n_neighbors = 30, min_dist = 0.3, umap.method = ‘‘umap-learn’’, metric = ‘‘correlation’’. These processing steps have been previously described (*31*) and are documented in jupyter notebooks as part of the COPILOT workflow.
To follow the developmental progression from the meristem to the elongation zone more closely, we updated the root atlas (*31*) developmental annotation to subdivide the meristem into the proliferation domain and transition domain as previously defined (*32*). The previous meristem annotation of the root atlas was based on correlation annotation by comparing each cell from scRNA-seq to bulk data from morphologically defined sections (*107*). On the other hand, HIGH PLOIDY2 was used to mark the meristem in a second bulk expression profile (*108*), which corresponds to the proliferation domain defined by Ivanov and Dubrovsky. Therefore, we leveraged correlation-based annotations derived from Li et al., 2016 to re-label the meristem of the atlas. If cells were defined as “meristem” by both Li et al., 2016 and Brady et al., 2007, then they were re-labeled as the proliferation domain. Those that were called elongation in the Li et al., 2016 annotation, but meristem in the Brady et al., 2007 annotation were re-labeled as the transition domain. Finally, cells labeled as elongation in both Brady and Li datasets but annotated as meristem in the root atlas were re-labeled as elongation zone.
Consistent with our annotation, we found that cell cycle-related genes were enriched in the proliferation domain of the atlas (fig. S2B), whereas *SMR1* (AT3G10525), a marker of endoreduplication, increased in the transition domain (*109*). The developmental annotation of cortex markers *CO2* (AT1G62500) and *CORTEX* (AT1G09750) also matched their expression patterns in the root (*110*, *111*).
We used the receiver operating characteristic (ROC) test implemented in Seurat FindMarkers to identify genes enriched in each developmental zone. A largely distinct set of markers was enriched in the transition domain (fig. S2C and Data S2). These included genes involved in vesicle-mediated transport (fig. S2D), in line with the observation that vesicle recycling activity is highest in this region (*112*).
We transferred the cell type and developmental stage labels from the wild-type atlas (*31*) to each sample using the Seurat label transfer workflow (*80*, *113*). To align corresponding cell types and developmental stages, we integrated samples from each experiment using the Seurat reference-based integration pipeline (*80*, *113*). A sample from the atlas with the highest genes detected (sc_12) was used as a reference (*31*) and two previously described samples (dc_1 and dc_2) (*34*) were also included in each integration. PCA and UMAP were subsequently calculated for each integration object using the batch-corrected “integrated” assay as described above. Although sc_12, dc_1, and dc_2 were not used in any subsequent analysis, their inclusion at the integration step helped to generate a comparable UMAP projection among different integration objects that facilitates interpretability.

Plotting gene expression values on the UMAP projection

We subsequently examined changes in cell state caused by the BL treatments or in the mutants profiled by plotting the log-normalized, ‘corrected’ counts produced by the SCTransform function (*106*) rather than the batch-corrected “integrated” values when visualizing changes in expression.

Pseudotime estimation and heatmaps of gene expression trends

Cortex cells were extracted from the integrated Seurat objects (brassinosteroid time course, *bri1-T* vs wild type and *gtl1 df1* vs wild type). Pseudotime was then inferred on the SCT assay of the extracted cortex cells using CytoTRACE v0.1.0(*114*). Once the pseudotime was calculated, the cortex cells were converted into SingleCellExperiment objects (*115*) before fitting a NB-GAM model (generalized additive model with a negative binomial distribution) using fitGAM function of tradeSeq R package v1.8.0 (*82*). The model-predicted expression trends were plotted with ComplexHeatmap in R (*116*)(v2.10.0).

Pseudobulk differential expression analysis

Benchmark studies point towards pseudobulk methods, which aggregate cell level counts for subpopulations of interest on a per-sample basis, as top performers for cross-condition comparisons in scRNA-seq (*81*, *117*). Therefore, we employed a pseudobulk approach implemented in muscat (Multi-sample multi-group scRNA-seq analysis tools) (*81*). We first performed differential expression analysis of our BL 2 hour vs BRZ samples after creating pseudobulk expression profiles for each sample, ignoring cell type and developmental stage labels, which is comparable to bulk RNA-seq profiling. This analysis identified 2,288 DEGs at the sample level, similar to previous bulk RNA-seq experiments in response to BL(*21*, *35*). To take advantage of the increased resolution afforded by scRNA-seq, we subsequently examined changes in each combination of cell type and developmental stage. Pseudobulk expression profiles were aggregated for each of these subpopulations by summing the raw counts using the aggregateData function. We then performed differential expression testing using the edgeR method (*118*) incorporated in the pbDS function. A term for the experimental batch and/or replicate was included in the contrast to adjust for potential batch effects. A gene was considered differentially expressed in a given subpopulation if the false discovery-rate adjusted p-value was <=0.05, absolute fold change was >=1.5 and detection frequency was >=10% in one of the conditions. Gene ontology enrichment analysis was conducted on the differentially expressed genes using the R package "gprofiler2" (*119*). Comparisons between DEG lists were performed using the GeneOverlap package (version 1.12.0; http://shenlab-sinai.github.io/shenlab-sinai/). p-values for intersections between gene lists were computed using Fisher's exact test. Visualizations were generated using Seurat (*80*), ComplexHeatmap (*116*), and ggplot2 (*120*).

WOT differential expression along cortex cell wall responsive trajectories

WOT constructs trajectories of cells from a reference time point by minimizing the difference over all genes (*41*). The algorithm requires as input the expression profiles of cells as well as an estimation of their proliferation rate. We estimated proliferation rates using imaging data (*121*), as previously described (*122*). As only the bottom 0.5 cm of each root was observed at each time, we expect some cells to exit the observed section due to proliferation. We estimated the number of cells that should exit the observed section based on the growth rate with the assumption that the section of root stays in equilibrium and assigned the calculated number of cells with the highest pseudotime a growth rate of zero so that they have no descendants in the observed root section at the next time point. We constructed trajectories using full gene expression profiles and evaluated the quality of the trajectories by checking the proportion of cells whose highest fate probability matched the annotation. We found that for 90% of cells, the largest fate assigned by WOT matched the annotation, rising to 97% in the maturation zone, where we have the greatest confidence in the annotation.

The cell wall signature was calculated for each cell by taking the sum of Z-scores for each of the 107 brassinosteroid-induced cell wall-related genes in the signature (GO:0071554), truncated to [-5,5]. We defined the cortex cell wall responsive subset as cortex cells with a cell wall score greater or equal to 1. This threshold was chosen as it selected less than 5% of cells from other lineages while still retaining >20% of cortex cells at the 2-hour time point. Any cortex cell that did not belong to the cortex responsive group was labeled as "cortex non-responsive". We performed differential expression on the cortex responsive versus non-responsive subsets at 2 hours, using WOT lineages to also perform differential expression on their putative ancestors and descendants. Statistical significance was evaluated using Welch's t-Test with adjusted p-values for multiple tests, requiring $t_{FDR} < 0.01$. Results were ranked by the adjusted expression ratio

$$P_{max}/(P_{min} + \varepsilon)$$

with $\varepsilon = 0.1$, where larger ε puts more emphasis on genes with non-zero expression in both groups. To generalize our WOT analysis we also constructed trajectories for each combination of cell type and developmental stage and performed differential expression analysis between each time point using the same process.

Gene regulatory networks

In order to construct GRNs, we used CellOracle (v0.7.0) for single-cell GRN inference (*60*). In the first stage of the CellOracle pipeline, a base GRN is defined, representing a global set of biologically plausible Transcription factor-Target interactions. We used publicly available scATAC-seq data from *Arabidopsis* roots (*123*) GSE155304:GSM4698760; (*123*) to determine regions of open chromatin. Cell Ranger ATAC (v1.2.0) was used to process raw scATAC seq data to call a peak-by-cell matrix. Cicero (v1.11.1) (*124*) was implemented to infer a co-accessibility map of chromatin regions. Transcription start sites were then annotated based on the *Arabidopsis* TAIR10 genome assembly. Finally, peaks with weak co-accessibility scores were filtered following instructions from CellOracle manual (https://morris-lab.github.io/CellOracle.documentation/tutorials/base_grn.html). To expand the number of transcription factors present in the base GRN we also included TF-Target interactions from DNA affinity purification sequencing (DAP-seq) (*125*) and a previously constructed integrative gene regulatory network (iGRN) (*126*). Our resulting base GRN contained 11.7 million interactions between 1,601 transcription factors and 31,019 target genes.

In the second step of the CellOracle pipeline, a regularized machine learning approach is used to define active edges and their regulatory strength in clusters or subpopulations of scRNA-seq data. In this process, the expression of target genes is predicted based on regulatory transcription factor levels from the base GRN. Inactive edges with low predictive ability are pruned from the base GRN, revealing context-specific GRN configurations (*60*).

To test CellOracle on *Arabidopsis* root data, we first inferred GRN configurations for each of the 36 cell type and developmental stage combinations in our WT atlas (*31*) using the SCT normalized counts. We limited the base GRN to genes dynamically expressed along pseudotime for each cell type plus associated transcription factors (*127*). Each cell type GRN was then constructed with default parameters following the CellOracle manual. To filter network edges with the "filter_links" function, we retained the top 20,000 edges (p-value <=0.01) for each subnetwork. This recovered known developmental regulators, including MYB36 in the endodermis (*128*) and BRN1/BRN2 in the root cap (*129*), confirming that CellOracle analysis of *Arabidopsis* root scRNA-seq data can infer GRNs configurations for particular cell identities and states.

We implemented similar procedures to infer context-specific GRN configurations for each cell type, developmental stage, and time point of the brassinosteroid time course samples (sc_43-50). We used transcription factors plus DEGs from BL 2 hour vs BRZ pseudobulk analysis of each cell type/developmental zone combination. The resulting set of 201 GRN configurations spanned 767,970 edges between 1,164 transcription factors and 7,135 targets. Network centrality measures were calculated using the built-in functions of the CellOracle pipeline.

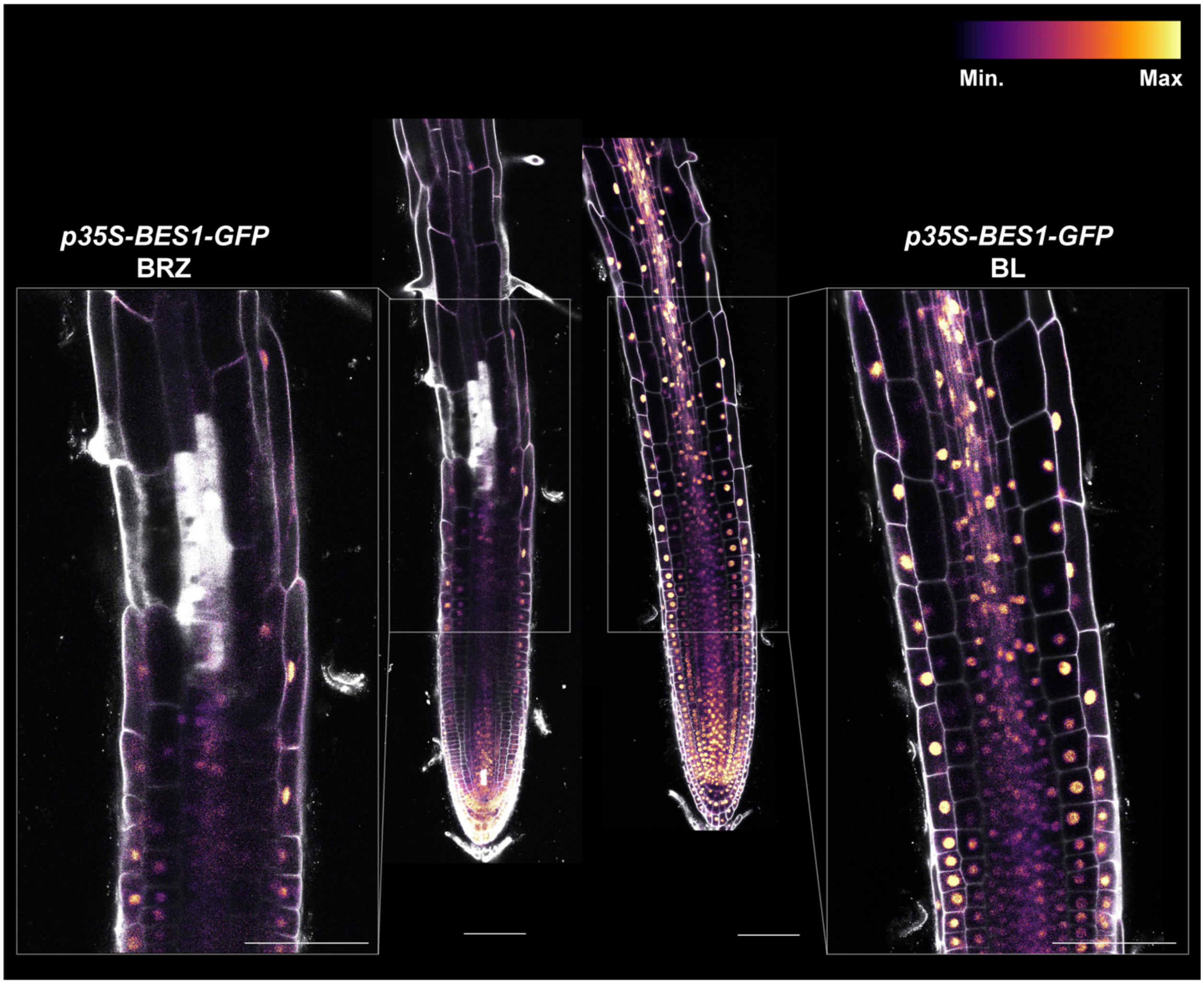

Fig. S1. A sensitized system to study spatiotemporal brassinosteroid response. Plants were grown for 7 days on 1 µM BRZ to deplete endogenous brassinosteroids, then transferred to either a fresh BRZ plate or 100 nM Brassinolide (BL) for 2 hours. p35S-BES1-GFP was used to monitor the efficacy of the sensitized system for BL scRNA-seq. BES1-GFP was predominantly present in the cytoplasm under low brassinosteroid conditions resulting from BRZ treatment but accumulated in the nucleus following BL treatment.

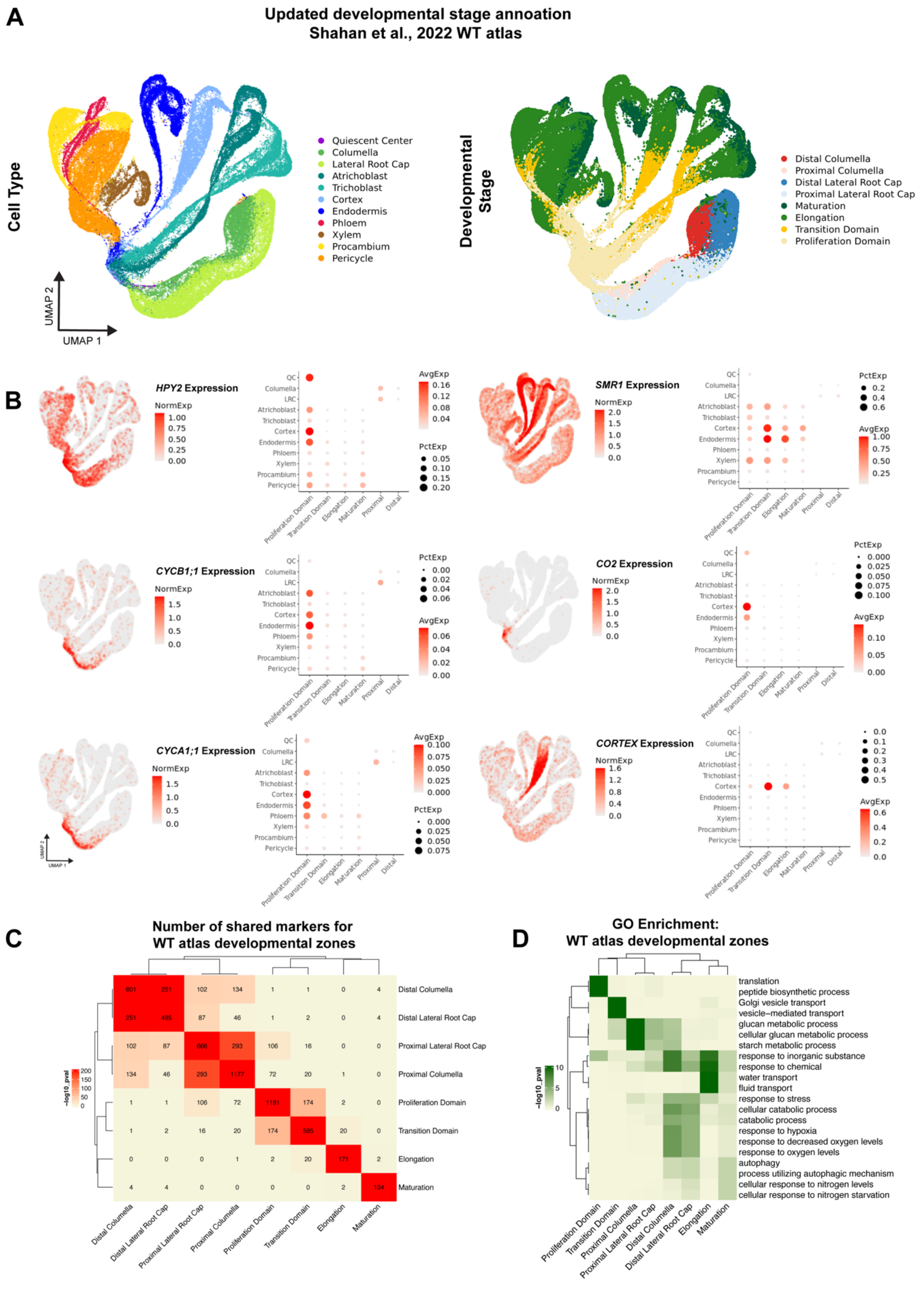
A
Updated developmental stage annoation
Shahan et al., 2022 WT atlas
Cell Type
Quiescent Center
Columella
Lateral Root Cap
Atrichoblast
Trichoblast
Cortex
Endodermis
Phloem
Xylem
Procambium
Pericycle
UMAP 2
UMAP 1
Developmental Stage
Distal Columella
Proximal Columella
Distal Lateral Root Cap
Proximal Lateral Root Cap
Maturation
Elongation
Transition Domain
Proliferation Domain
B
HPY2 Expression
SMR1 Expression
CYCB1;1 Expression
CO2 Expression
CYCA1;1 Expression
CORTEX Expression
NormExp
AvgExp
PctExp
QC
Columella
LRC
Atrichoblast
Trichoblast
Cortex
Endodermis
Phloem
Xylem
Procambium
Pericycle
Proliferation Domain
Transition Domain
Elongation
Maturation
Proximal
Distal
C
Number of shared markers for
WT atlas developmental zones
-log10_pval
Distal Columella
Distal Lateral Root Cap
Proximal Lateral Root Cap
Proximal Columella
Proliferation Domain
Transition Domain
Elongation
Maturation
D
GO Enrichment:
WT atlas developmental zones
translation
peptide biosynthetic process
Golgi vesicle transport
vesicle-mediated transport
glucan metabolic process
cellular glucan metabolic process
starch metabolic process
response to inorganic substance
response to chemical
water transport
fluid transport
response to stress
cellular catabolic process
catabolic process
response to hypoxia
response to decreased oxygen levels
response to oxygen levels
autophagy
process utilizing autophagic mechanism
cellular response to nitrogen levels
cellular response to nitrogen starvation

Fig. S2. Updated developmental annotation distinguishes between the proliferation domain and transition domain of the meristem. (A) Wild-type atlas of the *Arabidopsis* root from (*31*) showing updated developmental stage annotation in which the meristem is separated into the proliferation domain and transition domain. **(B)** Expression of markers in wild-type atlas supporting the developmental stage annotation. The color scale on the UMAP projection represents log normalized, corrected UMI counts. In dotplots, the size of the dot represents the percentage of cells in which the gene is expressed. **(C)** Comparison of the number of shared markers for each of the developmental zones in the wild-type atlas. Color represents −log10 p-values from the indicated overlaps calculated from Fisher's exact test by GeneOverlap. The number of genes in each intersection is indicated inside each box. **(D)** Enriched GO terms for markers of each developmental zone in the wild-type atlas.

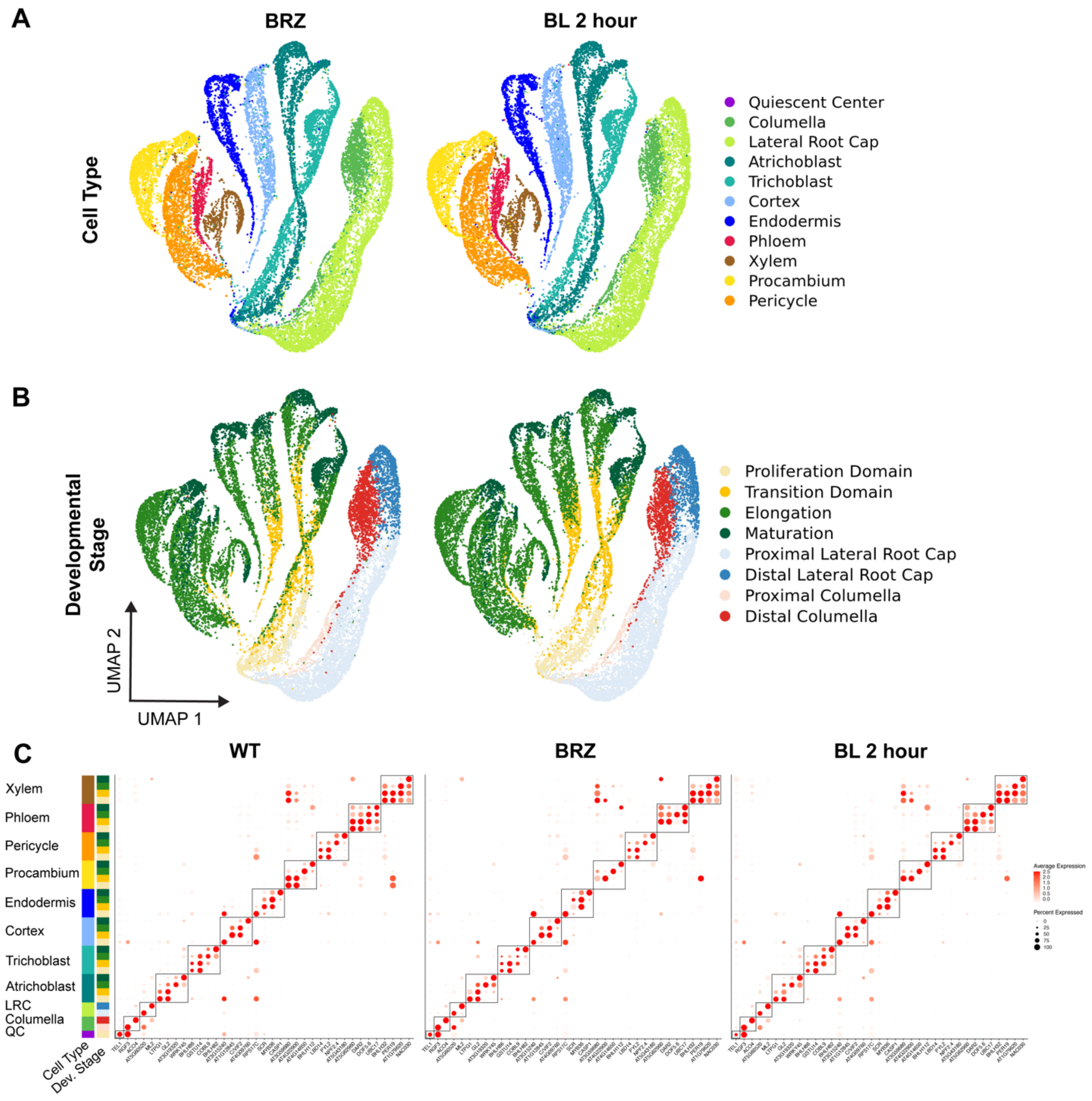

Fig. S3. Cell types and developmental stages are identified after label transfer in BL scRNA-seq. (A-B) Two-dimensional uniform manifold approximation and projection (UMAP) embedding of 21,473 BRZ and 22,275 two-hour BL treated cells across 3 biological replicates of scRNA-seq. Colors indicate (A) cell type or (B) developmental stage annotation. **(C)** Dotplots from the WT root altas (*31*), BRZ, and BL 2-hours scRNA-seq show that cell types and developmental stages are identified through label transfer. One marker gene for each cell type and developmental stage combination is shown. Circle size represents the percentage of cells in which a gene is expressed, and color represents the average expression level of each gene. Black boxes denote markers from each cell type. Colors of side annotations indicate cell type and developmental stage.

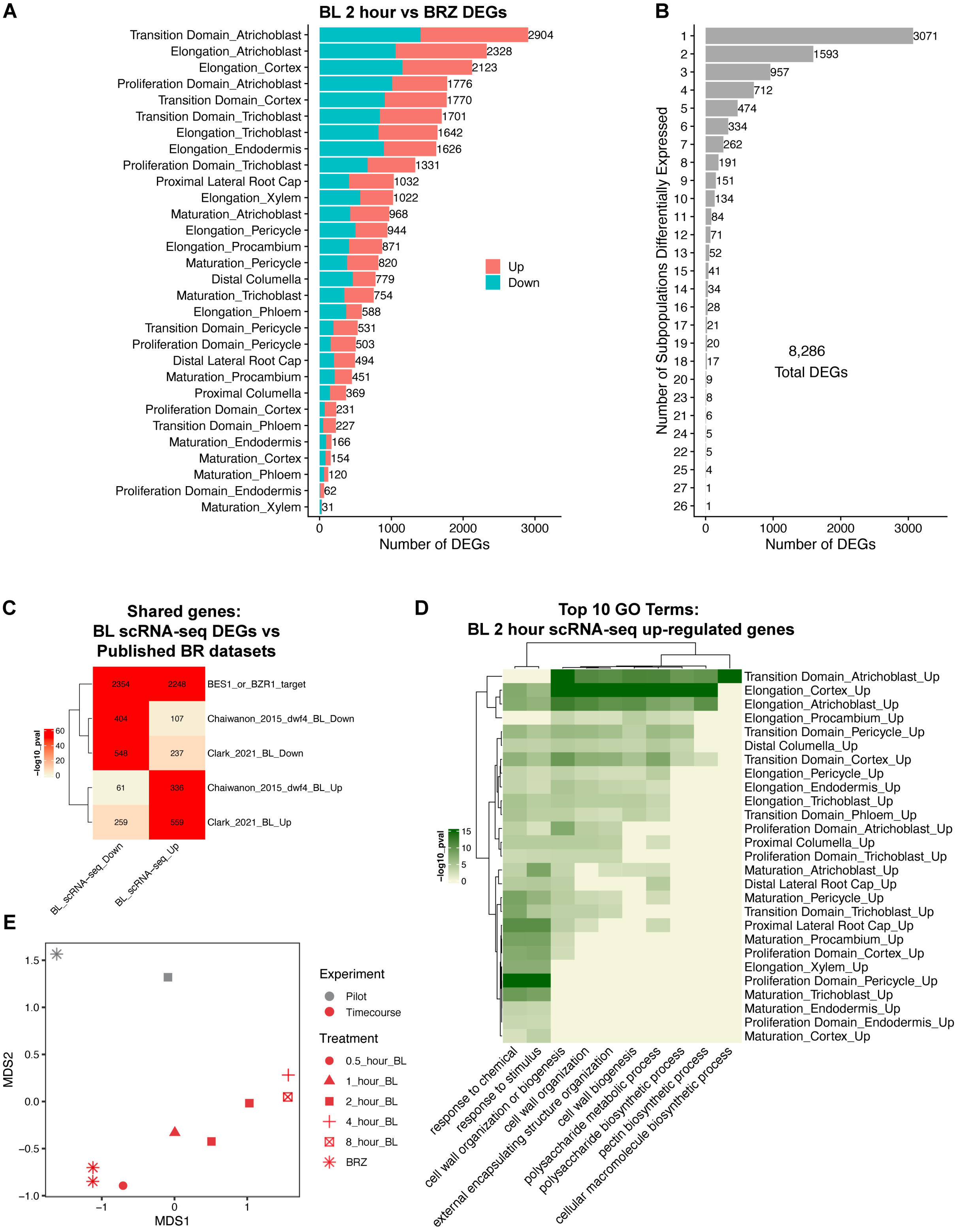
A
BL 2 hour vs BRZ DEGs
Transition Domain_Atrichoblast 2904
Elongation_Atrichoblast 2328
Elongation_Cortex 2123
Proliferation Domain_Atrichoblast 1776
Transition Domain_Cortex 1770
Transition Domain_Trichoblast 1701
Elongation_Trichoblast 1642
Elongation_Endodermis 1626
Proliferation Domain_Trichoblast 1331
Proximal Lateral Root Cap 1032
Elongation_Xylem 1022
Maturation_Atrichoblast 968
Elongation_Pericycle 944
Elongation_Procambium 871
Maturation_Pericycle 820
Distal Columella 779
Maturation_Trichoblast 754
Elongation_Phloem 588
Transition Domain_Pericycle 531
Proliferation Domain_Pericycle 503
Distal Lateral Root Cap 494
Maturation_Procambium 451
Proximal Columella 369
Proliferation Domain_Cortex 231
Transition Domain_Phloem 227
Maturation_Endodermis 166
Maturation_Cortex 154
Maturation_Phloem 120
Proliferation Domain_Endodermis 62
Maturation_Xylem 31
Up
Down
0 1000 2000 3000
Number of DEGs
B
Number of Subpopulations Differentially Expressed
1 3071
2 1593
3 957
4 712
5 474
6 334
7 262
8 191
9 151
10 134
11 84
12 71
13 52
15 41
14 34
16 28
17 21
19 20
18 17
20 9
23 8
21 6
24 5
22 5
25 4
27 1
26 1
8,286
Total DEGs
0 1000 2000 3000
Number of DEGs
C
Shared genes:
BL scRNA-seq DEGs vs
Published BR datasets
−log10_pval
60
40
20
0
2354 2248 BES1_or_BZR1_target
404 107 Chaiwanon_2015_dwf4_BL_Down
548 237 Clark_2021_BL_Down
61 336 Chaiwanon_2015_dwf4_BL_Up
259 559 Clark_2021_BL_Up
BL_scRNA−seq_Down
BL_scRNA−seq_Up
D
Top 10 GO Terms:
BL 2 hour scRNA-seq up-regulated genes
−log10_pval
15
10
5
0
Transition Domain_Atrichoblast_Up
Elongation_Cortex_Up
Elongation_Atrichoblast_Up
Elongation_Procambium_Up
Transition Domain_Pericycle_Up
Distal Columella_Up
Transition Domain_Cortex_Up
Elongation_Pericycle_Up
Elongation_Endodermis_Up
Elongation_Trichoblast_Up
Transition Domain_Phloem_Up
Proliferation Domain_Atrichoblast_Up
Proximal Columella_Up
Proliferation Domain_Trichoblast_Up
Maturation_Atrichoblast_Up
Distal Lateral Root Cap_Up
Maturation_Pericycle_Up
Transition Domain_Trichoblast_Up
Proximal Lateral Root Cap_Up
Maturation_Procambium_Up
Proliferation Domain_Cortex_Up
Elongation_Xylem_Up
Proliferation Domain_Pericycle_Up
Maturation_Trichoblast_Up
Maturation_Endodermis_Up
Proliferation Domain_Endodermis_Up
Maturation_Cortex_Up
response to chemical
response to stimulus
cell wall organization or biogenesis
cell wall organization
external encapsulating structure organization
cell wall biogenesis
polysaccharide metabolic process
polysaccharide biosynthetic process
pectin biosynthetic process
cellular macromolecule biosynthetic process
E
MDS2
1.5
1.0
0.5
0.0
−0.5
−1.0
−1 0 1
MDS1
Experiment
Pilot
Timecourse
Treatment
0.5_hour_BL
1_hour_BL
2_hour_BL
4_hour_BL
8_hour_BL
BRZ

Fig. S4. scRNA-seq identifies the elongating cortex as a site of BR-response. (A) Number of DEGs for each cell type/developmental stage combination in BL 2-hour scRNA-seq. Color indicates the number of up-regulated vs down-regulated genes. **(B)** The number of cell type/developmental stage combinations (subpopulations) in which each BL DEG is differentially expressed. 3,071/8,286 DEGs were altered in only a single sub-population. **(C)** Comparison of BL 2-hour DEGs from scRNA-seq to BES1 and BZR1 ChIP targets and previous bulk brassinosteroid RNA-seq datasets. Color represents −log10 p-values from the indicated overlaps calculated from Fisher's exact test by GeneOverlap. The number of genes in each intersection is indicated inside each box. **(D)** Top 10 GO terms in terms of p-value among BL up-regulated DEGs from scRNA-seq. Note the strong enrichment for cell wall-related GO terms in BL up-regulated genes in the elongating cortex. **(E)** Multi-dimensional scaling (MDS) plots based on sample-level pseudobulk counts from each BRZ or BL-treated scRNA-seq sample. Samples are arranged along MDS_1 according to treatment status. The BRZ and BL 2-hour samples from the pilot experiment that were processed and sequenced separately are distinct from the timecourse samples on MDS_2.

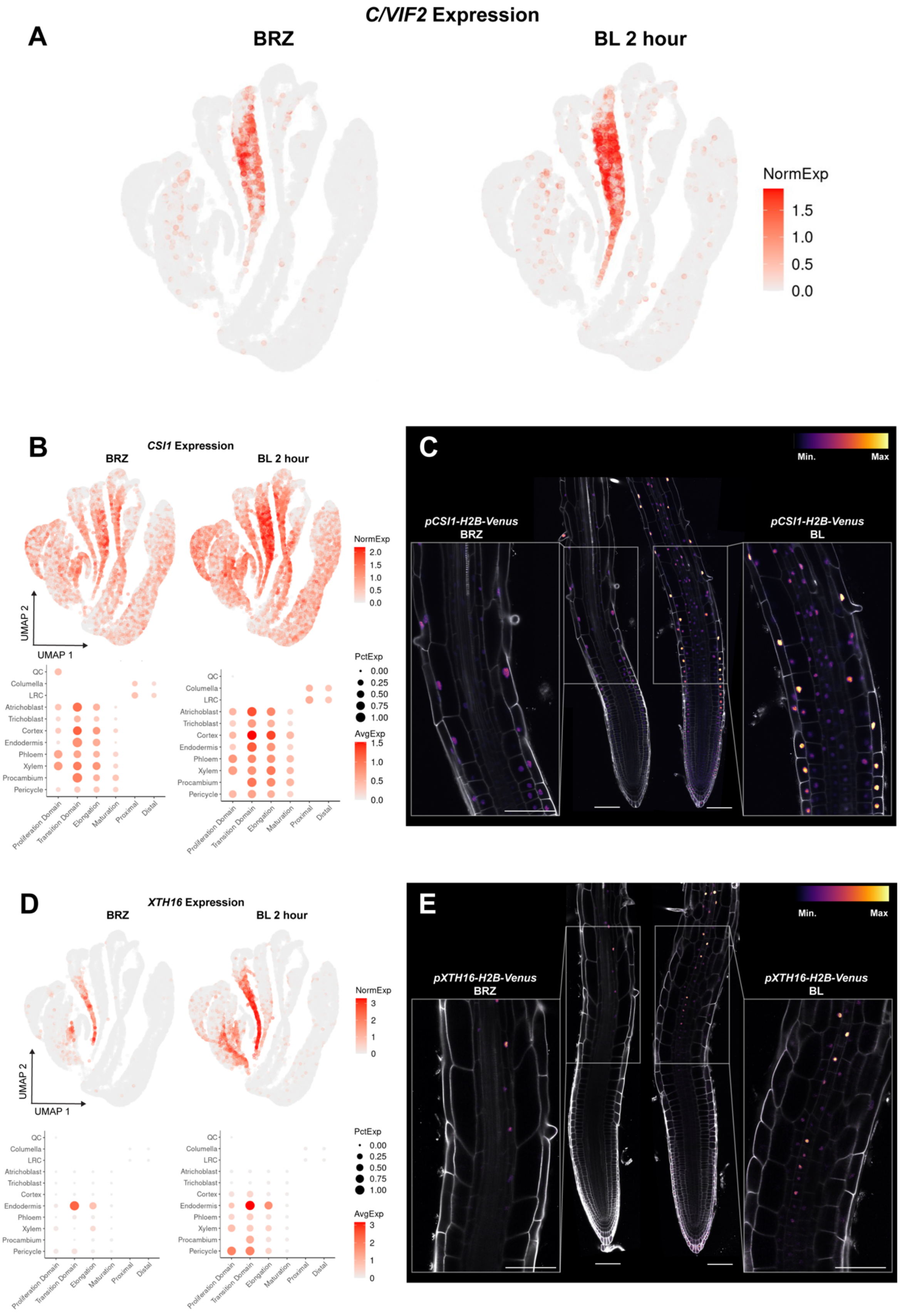
C/VIF2 Expression
A
BRZ
BL 2 hour
NormExp
1.5
1.0
0.5
0.0
B
CSI1 Expression
BRZ
BL 2 hour
NormExp
2.0
1.5
1.0
0.5
0.0
UMAP 2
UMAP 1
QC
Columella
LRC
Atrichoblast
Trichoblast
Cortex
Endodermis
Phloem
Xylem
Procambium
Pericycle
Proliferation Domain
Transition Domain
Elongation
Maturation
Proximal
Distal
PctExp
0.00
0.25
0.50
0.75
1.00
AvgExp
1.5
1.0
0.5
0.0
C
Min.
Max
pCSI1-H2B-Venus
BRZ
pCSI1-H2B-Venus
BL
D
XTH16 Expression
BRZ
BL 2 hour
NormExp
3
2
1
0
UMAP 2
UMAP 1
PctExp
0.00
0.25
0.50
0.75
1.00
AvgExp
3
2
1
0
E
Min.
Max
pXTH16-H2B-Venus
BRZ
pXTH16-H2B-Venus
BL

Fig. S5. Reporter gene expression is consistent with BL scRNA-seq. (A) Expression of *C/VIF2* in BRZ and BL 2-hour scRNA-seq. **(B)** *CSI1* expression in BRZ and BL 2 hour scRNA-seq. The color scale on the UMAP projection represents log normalized, corrected UMI counts. In dotplots, the size of the dot represents the percentage of cells in which the gene is expressed. **(C)** *CSI1-H2B-Venus* reporter grown on 1 μM BRZ for 7 days and transferred to 1 μM BRZ or 100 nM BL for 4 hours. Inset shows *CSI1* signals that are strongest in cortex and epidermis and increase with BL treatment. Propidium iodide-staining is shown in grey, with the color gradient indicating relative *CSI1-H2B-Venus* levels. **(D)** *XTH16* expression in BRZ and BL 2 hour scRNA-seq. **(E)** *XTH16-H2B-Venus* reporter grown on 1 μM BRZ for 7 days and transferred to 1 μM BRZ or 100 nM BL for 4 hours. Inset shows *XTH16* signals in the endodermis that increase with BL treatment. Propidium iodide-staining is shown in grey, with the color gradient indicating relative *XTH16-H2B-Venus* levels. Scale bars, 100 μm.

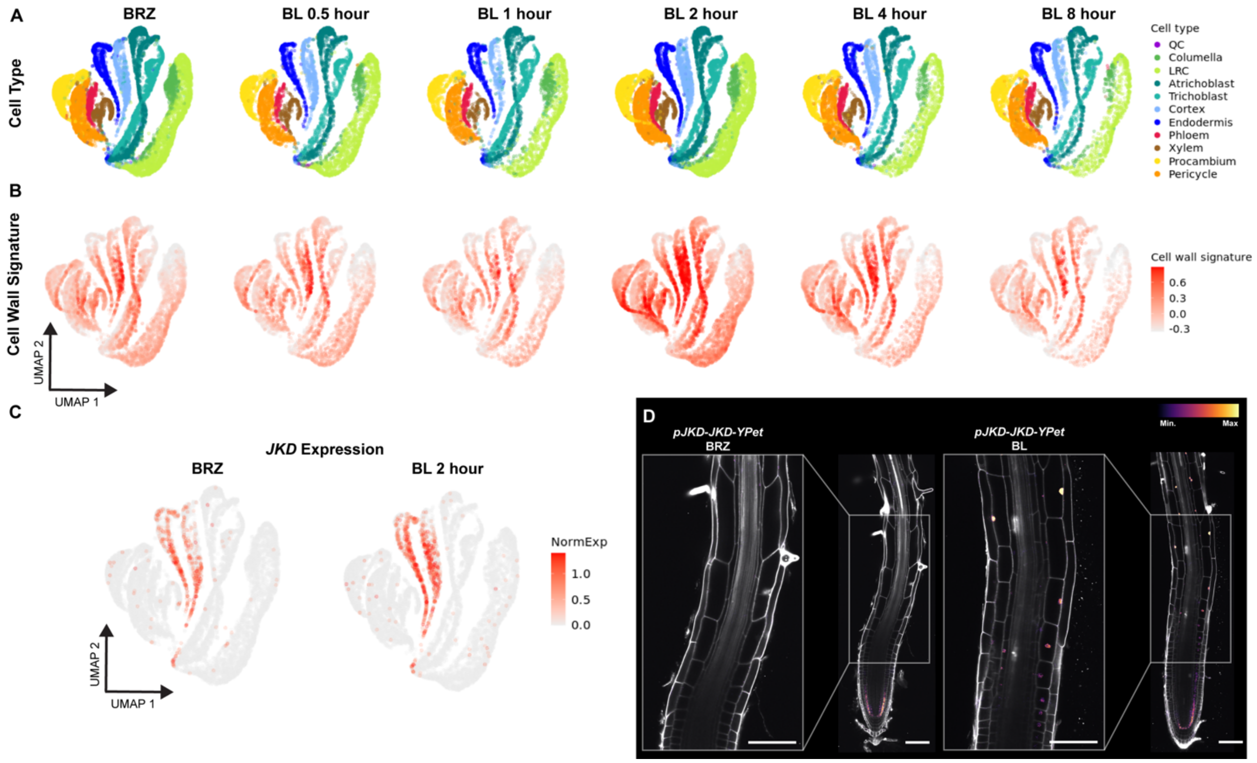

Fig S6. Waddington optimal transport identifies JKD as a brassinosteroid-responsive transcription factor along cortex trajectories. (A) UMAP showing cell type annotation across BL scRNA-seq treatment time course. This panel is repeated from the main text figure as a reference for the panel below. **(B)** UMAP projection colored by cell wall signature, calculated as the sum of Z-scores for each of the 107 brassinosteroid-induced cell wall-related genes in the signature (GO:0071554), truncated to [-5,5]. **(C)** Expression of *JKD* in BRZ and BL 2 hour scRNA-seq. The color scale represents log normalized, corrected UMI counts. **(D)** *pJKD-JKD-YPet* grown on 1 µM BRZ for 7 days and transferred to 1 µM BRZ or 100 nM BL for 4 hours. Inset shows *JKD* signals in the elongating cortex that increase with BL treatment. Propidium iodide-staining is shown in grey, with the color gradient indicating relative JKD-Ypet levels. Scale bars, 100 µm.

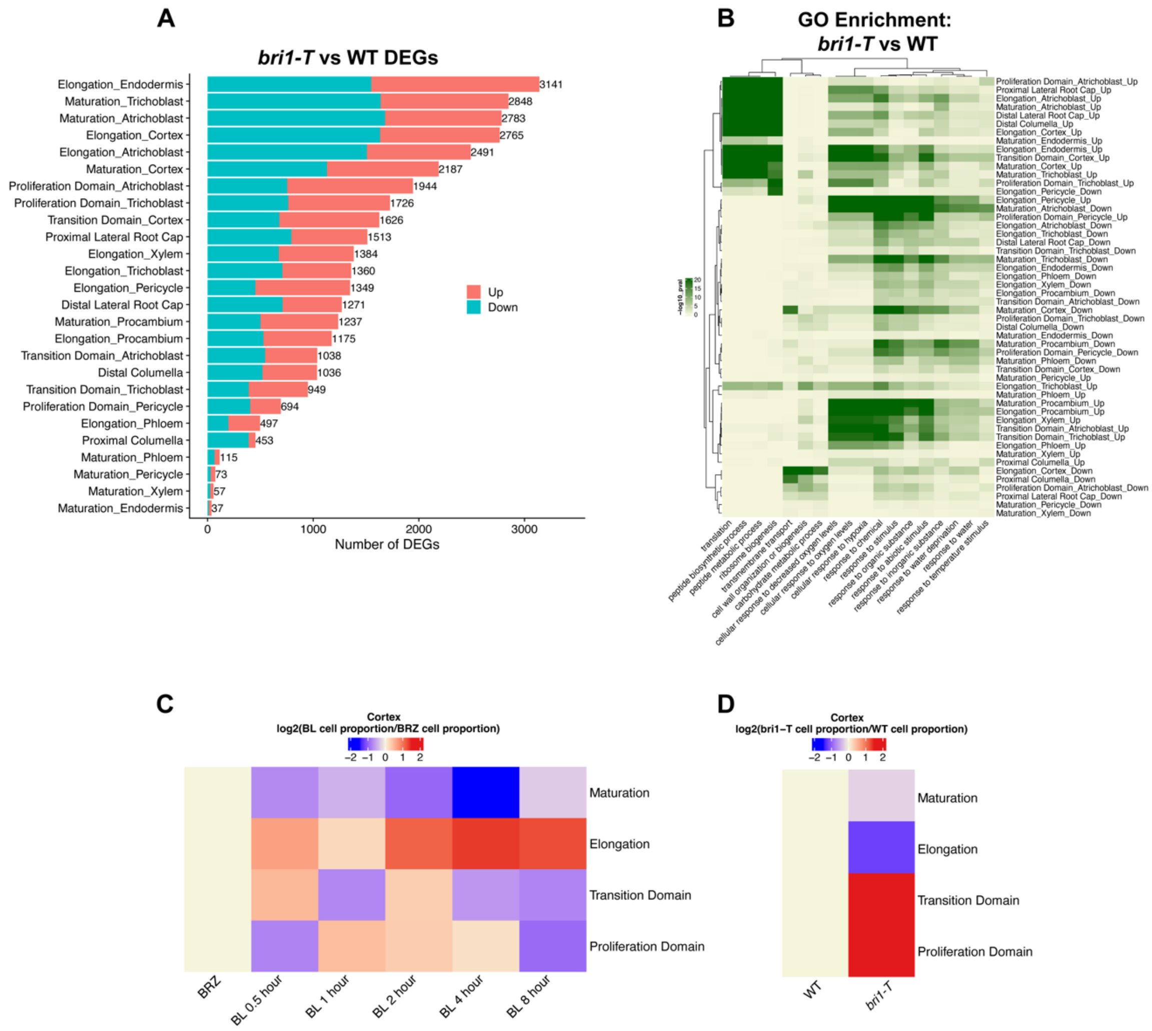

Fig. S7. Analysis of the triple receptor mutant *bri1-T* reveals endogenous regulation of cell-wall-related genes by brassinosteroids in the elongating cortex (A) Number of DEGs for each cell type/developmental stage combination in *bri1-T* scRNA-seq compared to WT. Color indicates the number of up-regulated vs down-regulated genes. **(B)** GO enrichment of *bri1-T* vs wild-type DEGs. Note the enrichment for cell wall-related GO terms in *bri1-T* down-regulated genes in the elongating cortex. **(C-D)** Heatmaps show an increased proportion of elongating cortex cells in response to BL (C), but a decreased proportion in *bri1-T* (D).

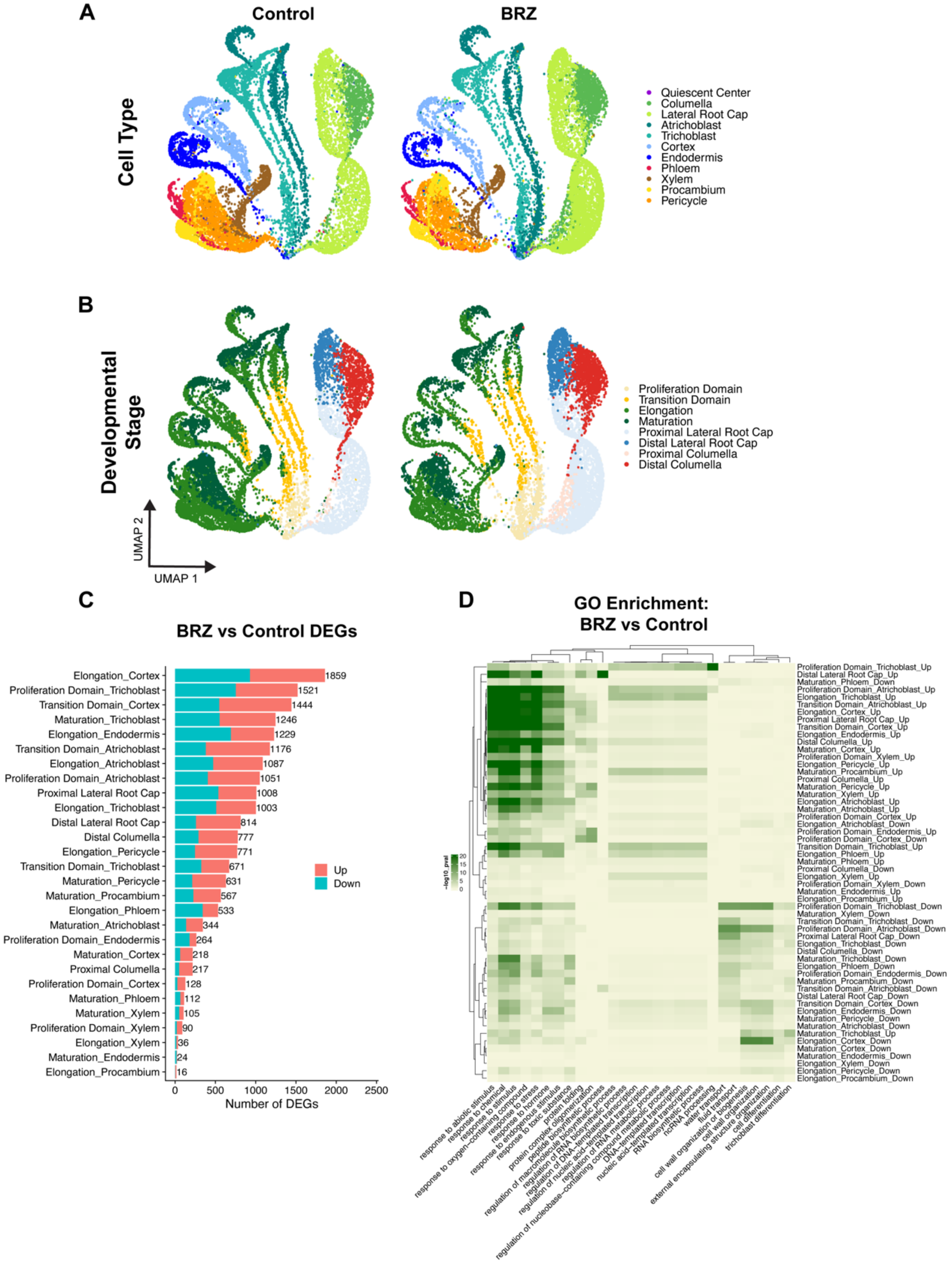
A
Control
BRZ
Cell Type
Quiescent Center
Columella
Lateral Root Cap
Atrichoblast
Trichoblast
Cortex
Endodermis
Phloem
Xylem
Procambium
Pericycle
B
Developmental Stage
Proliferation Domain
Transition Domain
Elongation
Maturation
Proximal Lateral Root Cap
Distal Lateral Root Cap
Proximal Columella
Distal Columella
UMAP 2
UMAP 1
C
BRZ vs Control DEGs
Elongation_Cortex 1859
Proliferation Domain_Trichoblast 1521
Transition Domain_Cortex 1444
Maturation_Trichoblast 1246
Elongation_Endodermis 1229
Transition Domain_Atrichoblast 1176
Elongation_Atrichoblast 1087
Proliferation Domain_Atrichoblast 1051
Proximal Lateral Root Cap 1008
Elongation_Trichoblast 1003
Distal Lateral Root Cap 814
Distal Columella 777
Elongation_Pericycle 771
Transition Domain_Trichoblast 671
Maturation_Pericycle 631
Maturation_Procambium 567
Elongation_Phloem 533
Maturation_Atrichoblast 344
Proliferation Domain_Endodermis 264
Maturation_Cortex 218
Proximal Columella 217
Proliferation Domain_Cortex 128
Maturation_Phloem 112
Maturation_Xylem 105
Proliferation Domain_Xylem 90
Elongation_Xylem 36
Maturation_Endodermis 24
Elongation_Procambium 16
Up
Down
0 500 1000 1500 2000 2500
Number of DEGs
D
GO Enrichment: BRZ vs Control
-log10_pval
20 15 10 5 0
response to abiotic stimulus
response to chemical
response to stimulus
response to oxygen-containing compound
response to stress
response to hormone
response to endogenous stimulus
response to toxic substance
protein folding
protein complex oligomerization
peptide biosynthetic process
regulation of macromolecule biosynthetic process
regulation of RNA biosynthetic process
regulation of DNA-templated transcription
regulation of nucleic acid-templated transcription
regulation of nucleobase-containing compound metabolic process
regulation of RNA metabolic process
DNA-templated transcription
nucleic acid-templated transcription
RNA biosynthetic process
ncRNA processing
water transport
fluid transport
cell wall organization or biogenesis
cell wall organization
external encapsulating structure organization
cell differentiation
trichoblast differentiation
Proliferation Domain_Trichoblast_Up
Distal Lateral Root Cap_Up
Maturation_Phloem_Down
Proliferation Domain_Atrichoblast_Up
Elongation_Trichoblast_Up
Transition Domain_Atrichoblast_Up
Elongation_Cortex_Up
Proximal Lateral Root Cap_Up
Transition Domain_Cortex_Up
Elongation_Endodermis_Up
Distal Columella_Up
Maturation_Cortex_Up
Proliferation Domain_Xylem_Up
Elongation_Pericycle_Up
Maturation_Procambium_Up
Proximal Columella_Up
Maturation_Pericycle_Up
Maturation_Xylem_Up
Elongation_Atrichoblast_Up
Maturation_Atrichoblast_Up
Proliferation Domain_Cortex_Up
Elongation_Atrichoblast_Down
Proliferation Domain_Endodermis_Up
Proliferation Domain_Cortex_Down
Transition Domain_Trichoblast_Up
Elongation_Phloem_Up
Maturation_Phloem_Up
Proximal Columella_Down
Elongation_Xylem_Up
Proliferation Domain_Xylem_Down
Maturation_Endodermis_Up
Elongation_Procambium_Up
Proliferation Domain_Trichoblast_Down
Maturation_Xylem_Down
Transition Domain_Trichoblast_Down
Proliferation Domain_Atrichoblast_Down
Proximal Lateral Root Cap_Down
Elongation_Trichoblast_Down
Distal Columella_Down
Maturation_Trichoblast_Down
Elongation_Phloem_Down
Proliferation Domain_Endodermis_Down
Maturation_Procambium_Down
Transition Domain_Atrichoblast_Down
Distal Lateral Root Cap_Down
Transition Domain_Cortex_Down
Elongation_Endodermis_Down
Maturation_Pericycle_Down
Maturation_Atrichoblast_Down
Maturation_Trichoblast_Up
Elongation_Cortex_Down
Maturation_Cortex_Down
Maturation_Endodermis_Down
Elongation_Xylem_Down
Elongation_Pericycle_Down
Elongation_Procambium_Down

Fig. S8. BRZ scRNA-seq confirms endogenous regulation of cell-wall-related genes by brassinosteroids in the elongating cortex (A-B) Two-dimensional uniform manifold approximation and projection (UMAP) embedding of 16,642 Control and 14,320 BRZ treated cells across 2 biological replicates of scRNA-seq. Colors indicate (A) cell type or (B) developmental stage annotation. **(C)** Number of DEGs for each cell type/developmental stage combination in WT BRZ scRNA-seq compared to WT control. Color indicates the number of up-regulated vs down-regulated genes. **(D)** GO enrichment of BRZ vs control DEGs. Note the enrichment for cell wall-related GO terms in BRZ down-regulated genes in the elongating cortex.

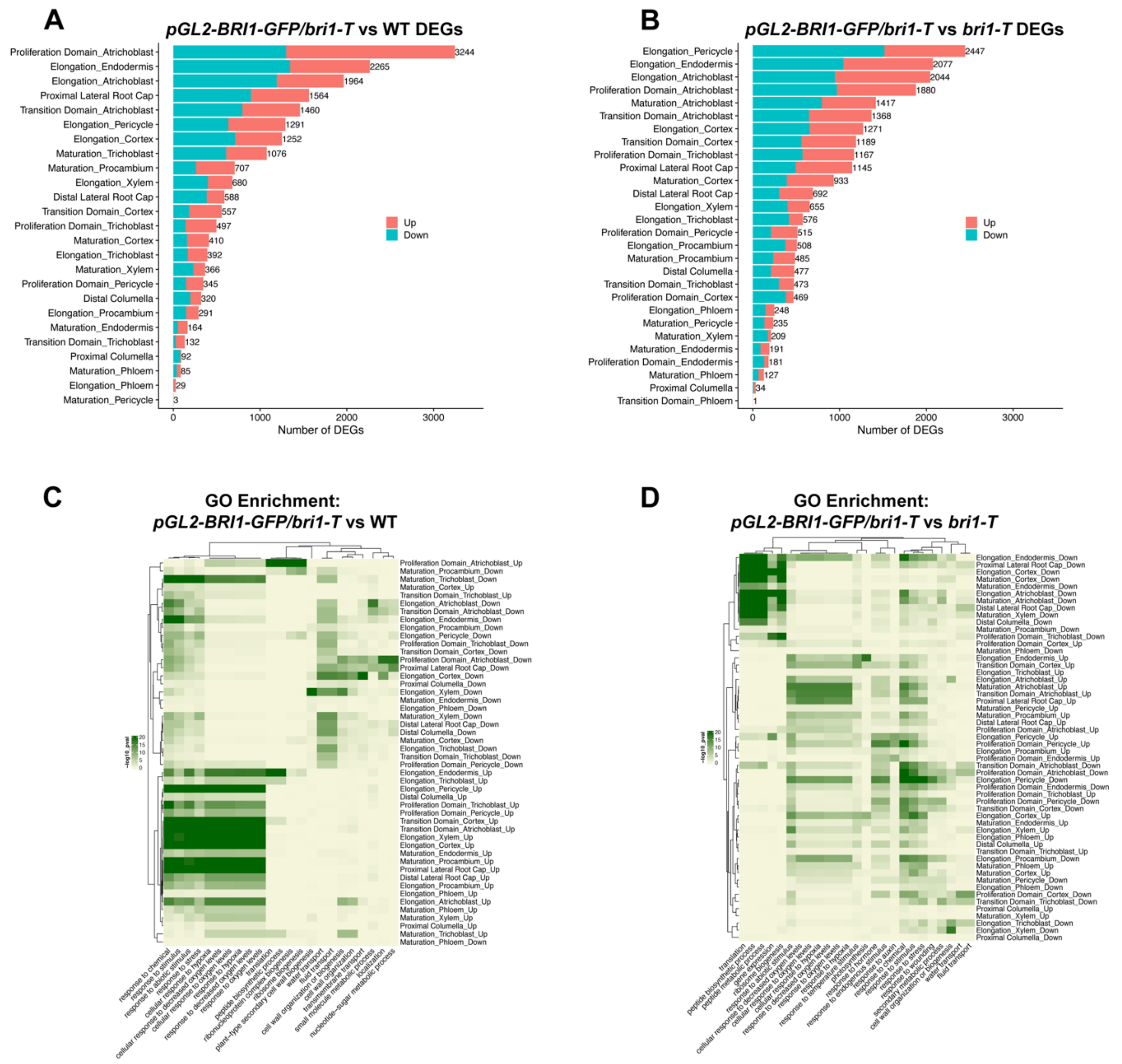

Fig. S9. Differentially expressed genes in *pGL2-BRI1-GFP/bri1-T* scRNA-seq.

(A-B) Number of DEGs for each cell type/developmental stage combination in *pGL2-BRI1-GFP/bri1-T* scRNA-seq compared to WT (A) or *pGL2-BRI1-GFP/bri1-T* scRNA-seq compared to *bri1-T* (B). Color indicates the number of up-regulated vs down-regulated genes. **(C-D)** GO enrichment of DEGs in *pGL2-BRI1-GFP/bri1-T* vs wild-type (C) or *pGL2-BRI1-GFP/bri1-T* vs *bri1-T* (D).

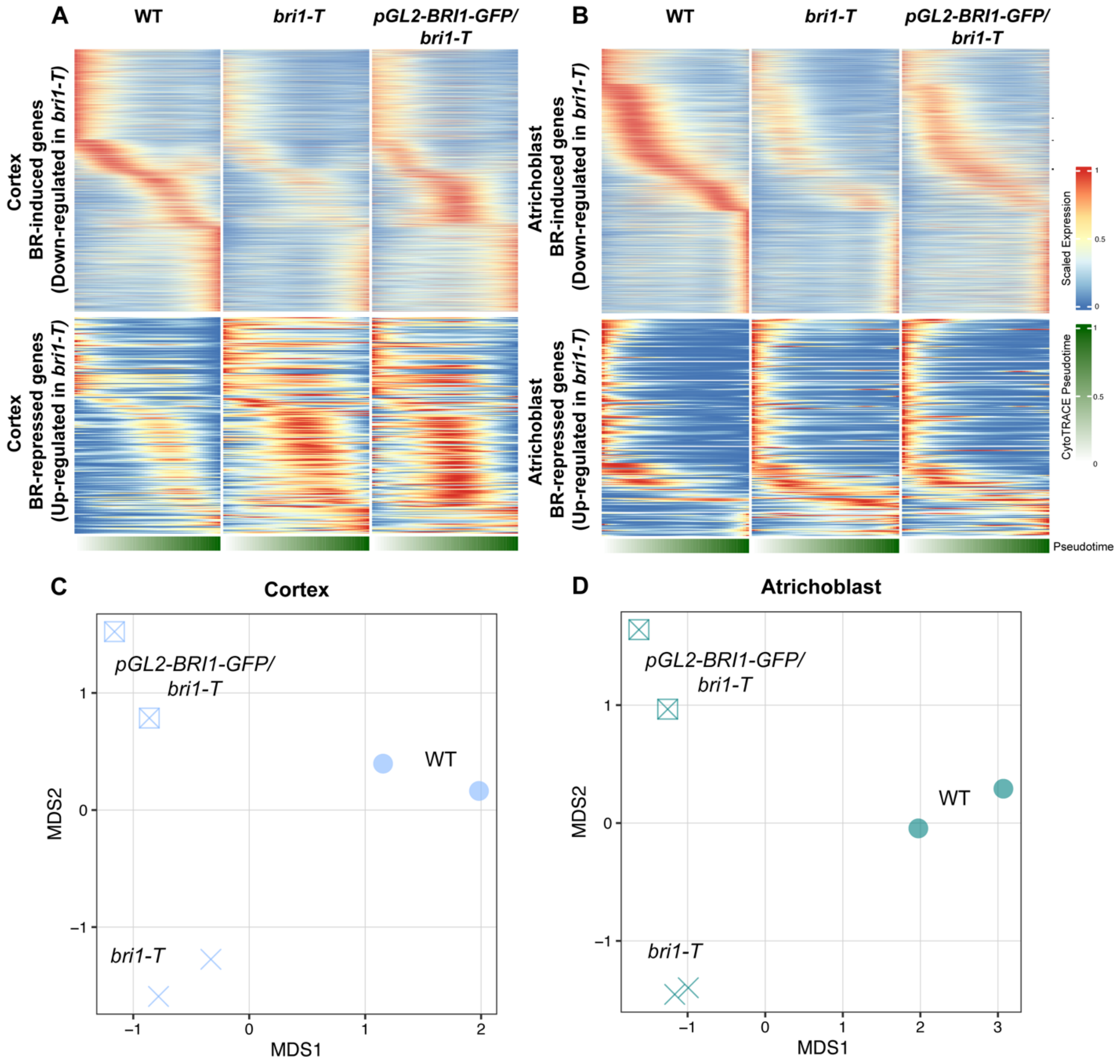

Fig. S10. Distinct gene expression patterns in *pGL2-BRI1-GFP/bri1-T.* (A-B) Gene expression trends for *bri1-T* vs wild-type DEGs along cortex (A) or atrichoblast (B) trajectories for WT, *bri1-T,* and *pGL2-BRI1-GFP/bri1-T*. Scaled expression along cortex pseudotime is plotted for each genotype. Lower bar indicates pseudotime progression calculated by CytoTRACE. **(C-D)** Multi-dimensional scaling (MDS) analysis of cortex (C) or atrichoblast (D) cells from scRNA-seq. Note that replicates from the same genotype group together, but genotypes are well separated.

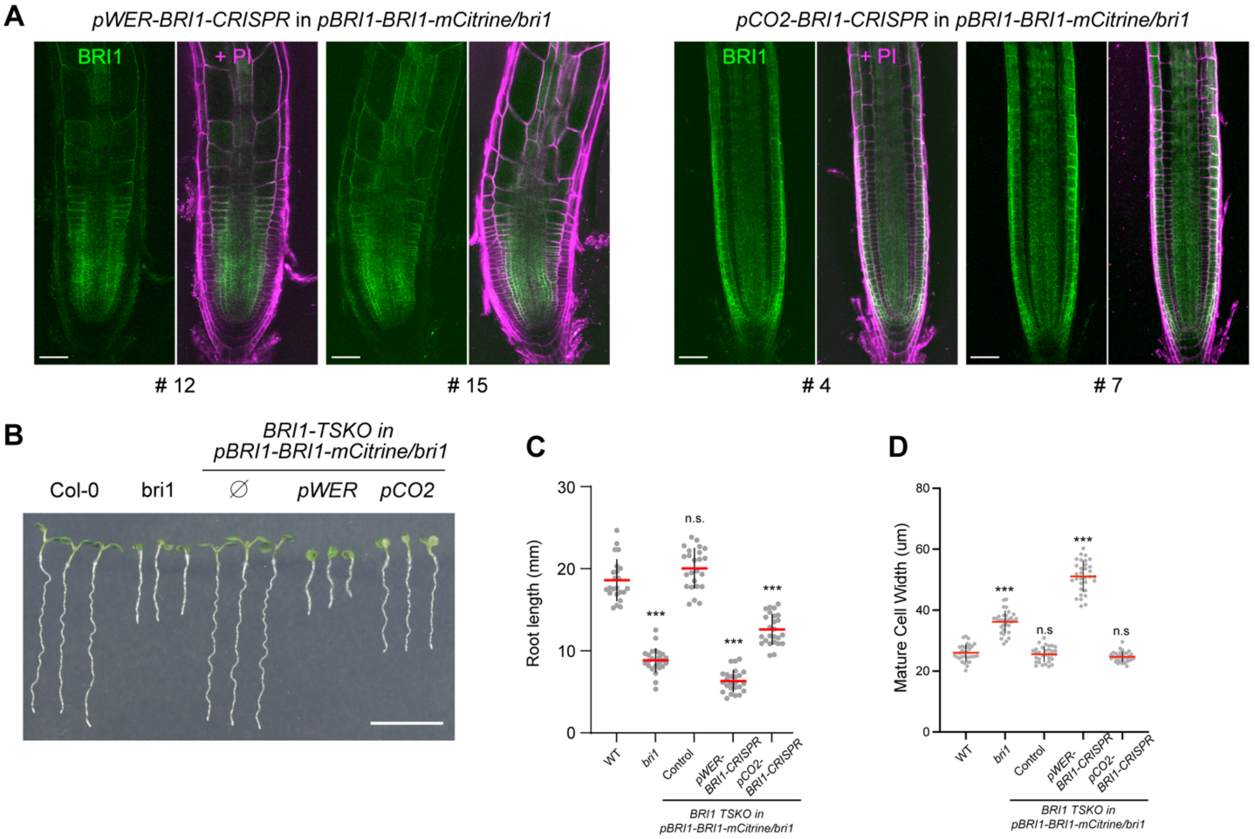

Fig. S11. ***BRI1*** **tissue-specific CRISPR. (A)** Two individual transgenic lines for *pWER-BRI1-CRISPR* and *pCO2-BRI1-CRISPR* exhibiting similar BRI1-mCitrine expression patterns. Scale bars, 50 µm. **(B)** Seven-day-old *BRI1-CRISPR* transgenic seedlings with roots shorter than those of the wild-type (Col-0) control and complemented *pBRI1-BRI1-mCitrine/bri1*. Scale bar represents 1 cm. **(C)** Quantification of the root length of transgenic lines shown in (B). **(D)** Quantification of the mature cortex cell widths. All individual data points are plotted. Red horizontal bars represent the means, and error bars represent s.d. Significant differences between transgenic lines and the WT control were determined by one-way ANOVA and Dunnett's multiple comparisons tests. ***$P < 0.001$, **$P < 0.01$, *$P < 0.05$. n.s. not significant.

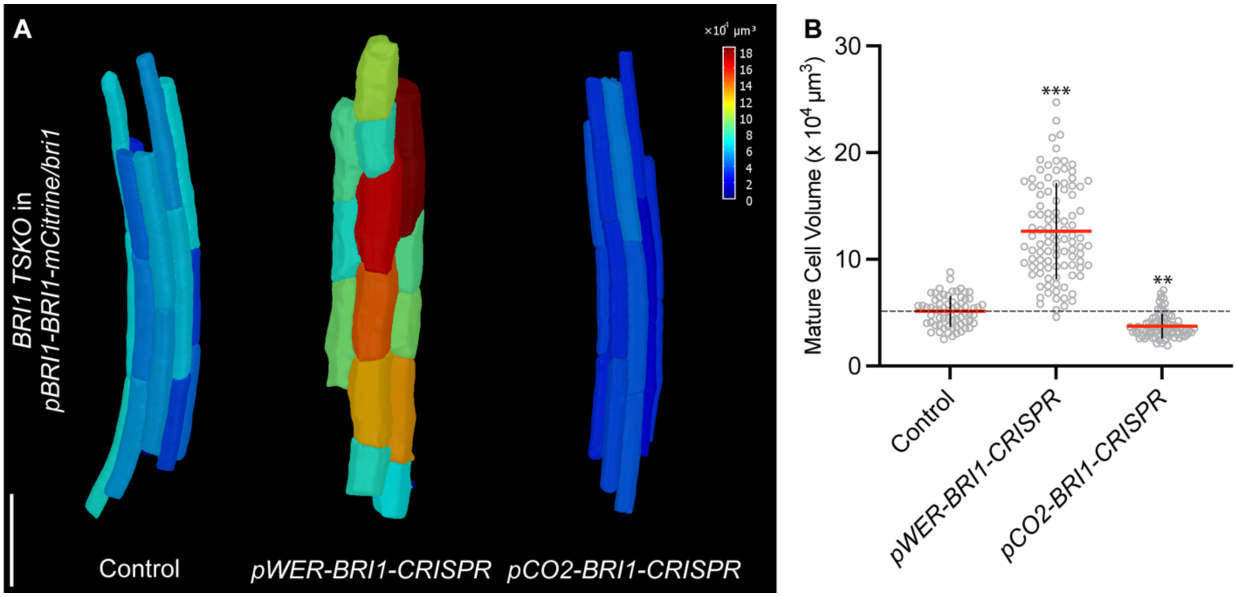

Fig. S12. Analysis of cortex cell volume in BRI1 TSKO mutants (A) Representative mature cortex cell layer volume segmentations of *pBRI1-BRI1-mCitrine*/*bri1*, indicated as control, and *pWER-BRI1-CRISPR* and *pCO2-BRI1-CRISPR* transgenic lines, where *BRI1* was knocked out in epidermis and cortex cell layer, respectively. Cell volumes are visualized as heatmaps. Scale bar, 100 µm. **(B)** Quantification of cell volumes in (A) All individual data points are plotted. Red horizontal bars represent the means, and error bars represent s.d. Significant differences between CRISPR lines and the *pBRI1-BRI1-mCitrine*/*bri1* control were determined by one-way ANOVA and Dunnett's multiple comparisons tests. ***$P < 0.001$, **$P < 0.01$, *$P < 0.05$. n.s. not significant.

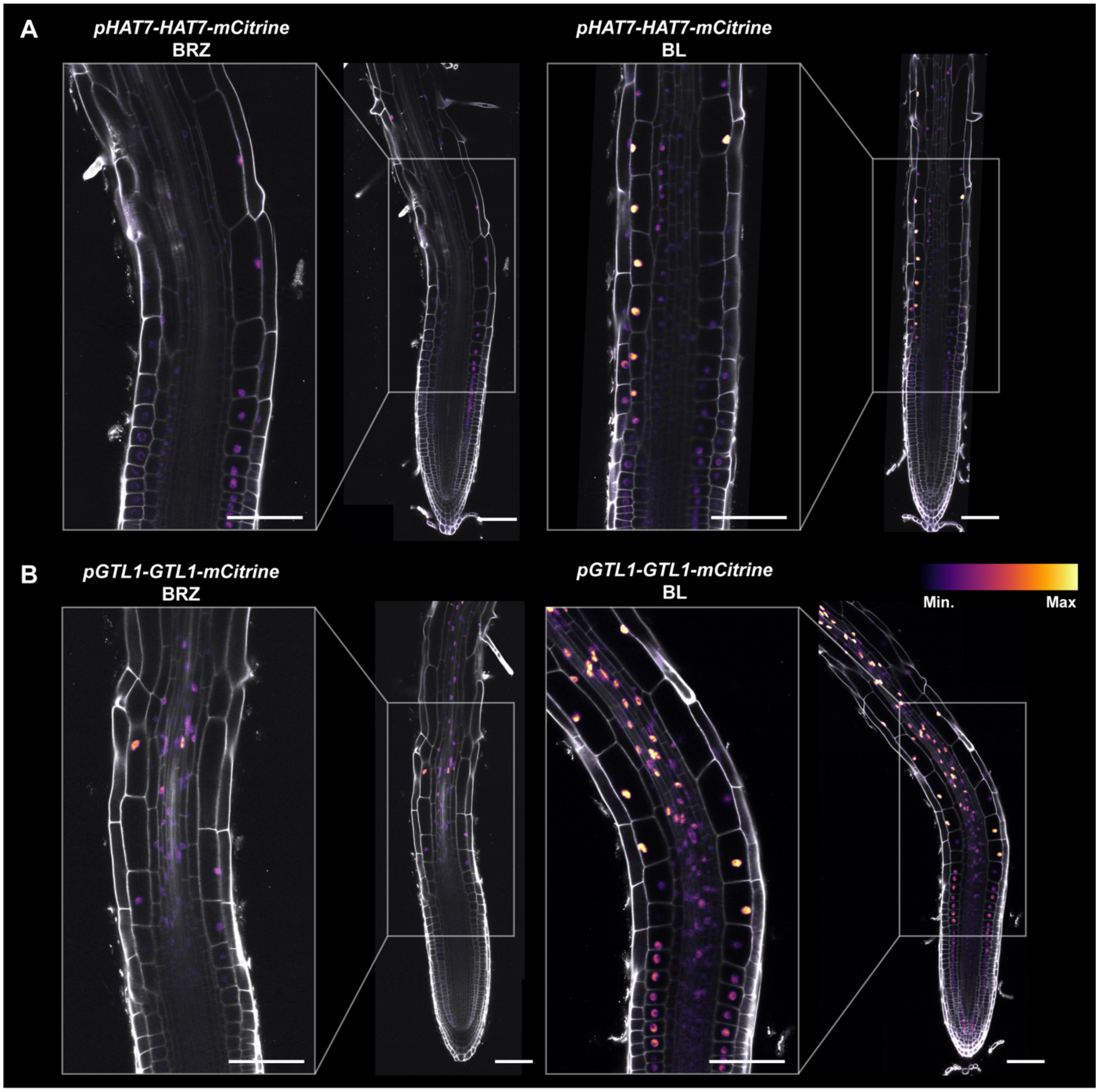

Fig. S13. ***HAT7* and *GTL1* are induced by BL treatment (A-B)** 7-day-old roots expressing *pHAT7-HAT7-mCitrine* (A) or *pGTL1-GTL1-mCitrine* (B) reporters under the indicated treatments. Plants were grown on 1 µM BRZ for 7 days and transferred to 1 µM BRZ or 100 nM BL for 4 hours. Propidium iodide-staining is shown in grey, with the color gradient indicating relative mCitrine levels. Scale bars, 100 µm.

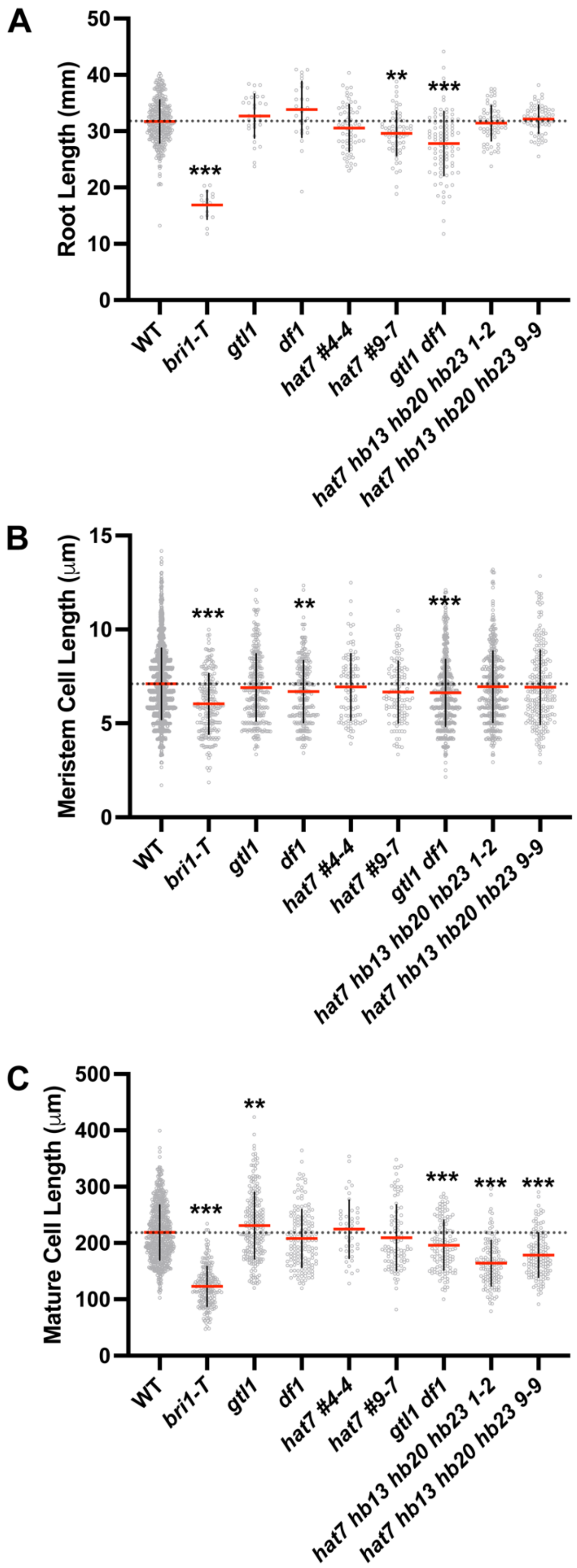

Fig. S14. ***HAT7* and *GTL1* family TFs affect cortex cell elongation. (A)** Quantification of the root length in the indicated mutants. *hat7 hb13 hb20 hb23 1-2* and *hat7 hb13 hb20 hb23 9-9* represent two independent CRISPR mutants. *hat7 hb13 hb20 hb23 1-2* is used as a representative allele throughout this study unless otherwise indicated. **(B)** Quantification of meristematic cortex cell length, defined as the first 20 cells of individual roots starting from the quiescent center. **(C)** Quantification of mature cortex cell length. All individual data points are plotted. Red horizontal bars represent the means, and error bars represent s.d. Significant differences between mutants and the wild-type control were determined by one-way ANOVA and Dunnett's multiple comparisons tests. ***$P < 0.001$, **$P < 0.01$, *$P < 0.05$.

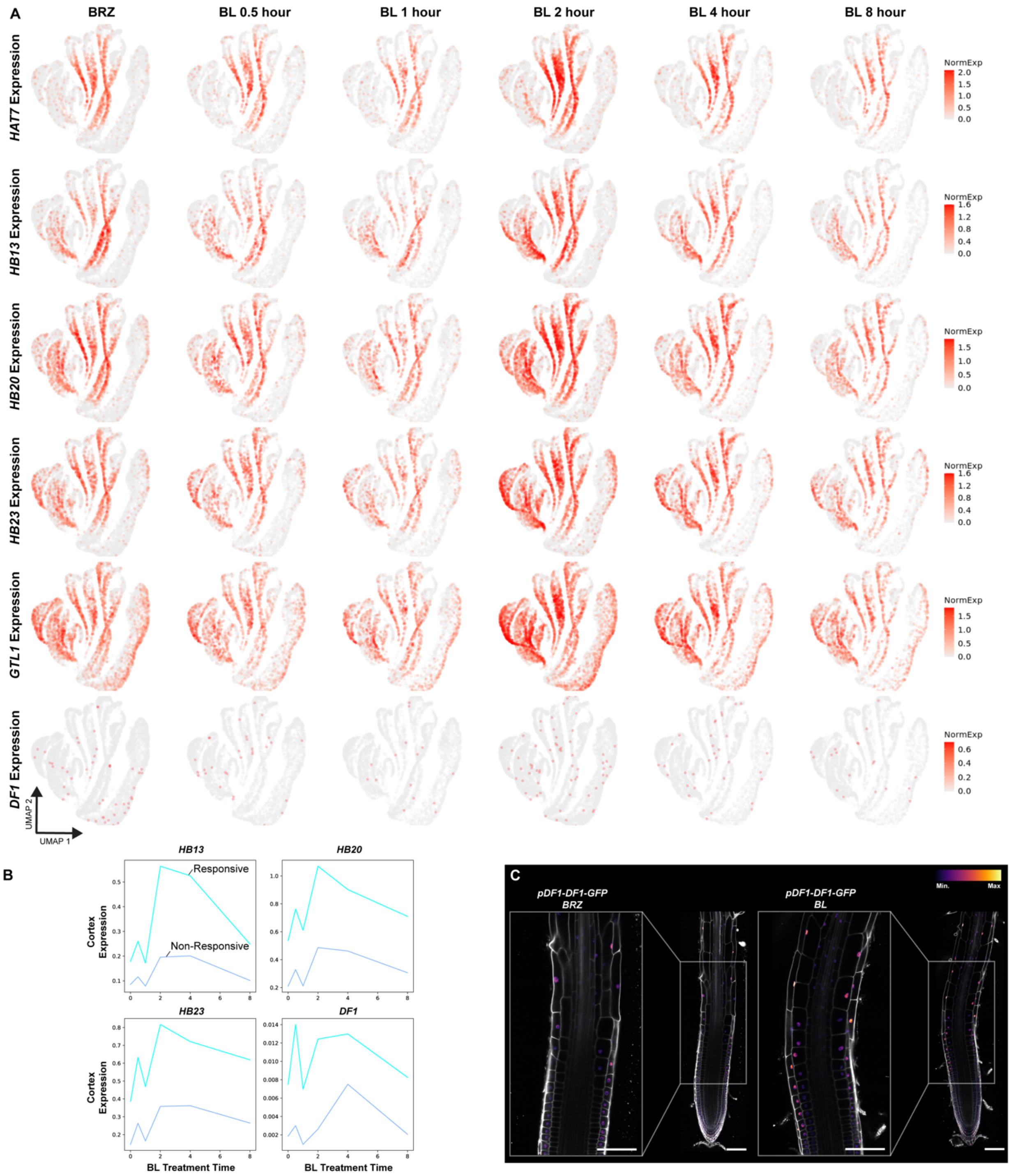

A
BRZ
BL 0.5 hour
BL 1 hour
BL 2 hour
BL 4 hour
BL 8 hour
HAT7 Expression
HB13 Expression
HB20 Expression
HB23 Expression
GTL1 Expression
DF1 Expression
NormExp
UMAP 2
UMAP 1
B
HB13
HB20
HB23
DF1
Responsive
Non-Responsive
Cortex Expression
BL Treatment Time
C
pDF1-DF1-GFP BRZ
pDF1-DF1-GFP BL
Min.
Max

Fig. S15. *HAT7* and *GTL1* family transcription factors are brassinosteroid-responsive regulators along cortex trajectories. (A) UMAP projections showing expression levels of HAT7 and GTL1 family transcription factors over the brassinosteroid time series scRNA-seq experiment. The color scale represents log normalized, corrected UMI counts. **(B)** Expression trends for indicated transcription factors along WOT cortex responsive vs cortex non-responsive trajectories. **(C)** *pDF1-DF1-GFP* grown on 1 µM BRZ for 7 days and transferred to 1 µM BRZ or 100 nM BL for 4 hours. Inset shows *DF1* signals in the elongating epidermis and cortex that increase with BL treatment. Propidium iodide-staining is shown in grey, with the color gradient indicating relative DF1-GFP levels. Scale bars, 100 µm.

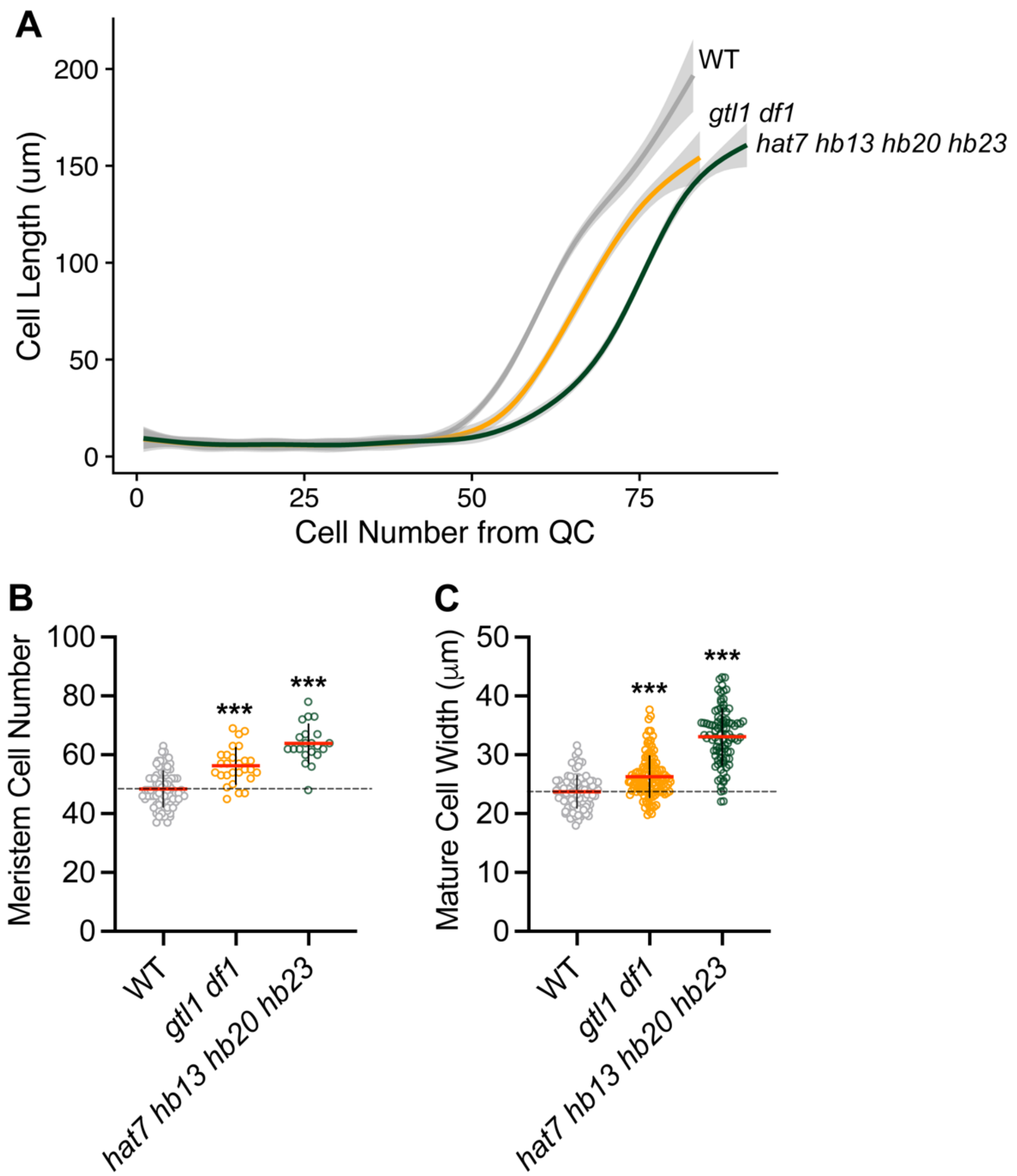

Fig. S16. Phenotypic analysis of *gtl1 df1* and *hat7 hb13 hb20 hb23* roots.

(A) Quantification of cortex cell length along the longitudinal axis of the root. The quiescent center was designated as "0" and each cell number consecutively thereafter. The grey area represents the confidence interval of the smoothed mean estimated with a generalized additive model. Number of roots per genotype: WT=51, *gtl1 df1*=26, *hat7 hb13 hb20 hb23*=16. **(B)** Quantification of meristem cell number as indicated by the first elongated cortex cell. **(C)** Quantification of mature cortex cell width. Red horizontal bars represent the means, and error bars represent s.d. Significant differences between each line and wild type were determined by one-way ANOVA and Dunnett's multiple comparison tests. ***$P < 0.001$.

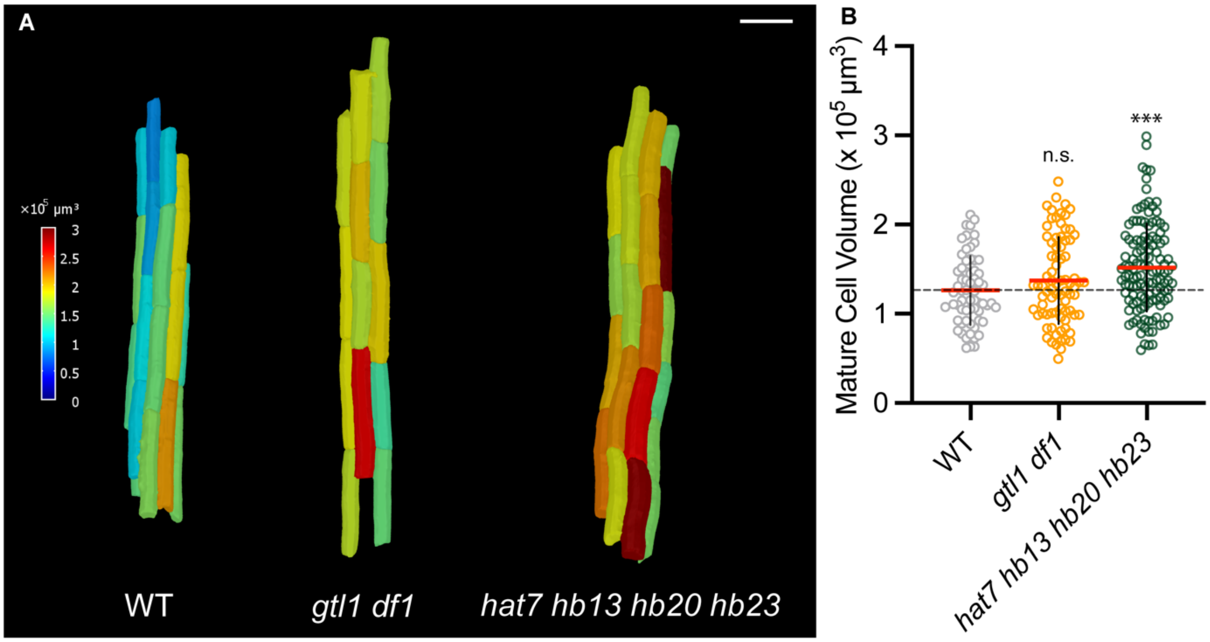

Fig. S17. Analysis of cortex cell volume in *gtl1 df1* and *hat7 hb13 hb20 hb23*.

(A) Representative mature cortex cell layer volume segmentations of wild-type, *gtl1 df1*, and *hat7 hb13 hb20 hb23*. Cell volumes are visualized as heatmaps. Scale bar, 100 µm. **(B)** Quantification of cell volumes in (A) All individual data points are plotted. Red horizontal bars represent the means, and error bars represent s.d. Significant differences between mutants and the wild-type control were determined by one-way ANOVA and Dunnett's multiple comparisons tests. ***P < 0.001, **P < 0.01, *P < 0.05. n.s. not significant.

A

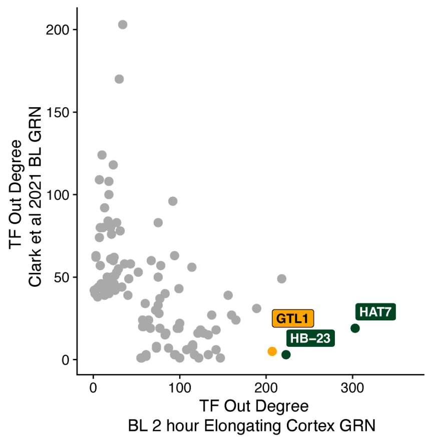

B

		BL 2 Hour Elongating Cortex GRN		Clark et al 2021 BL GRN	
Gene	Name	TF Rank	TF Out Degree	TF Rank	TF Out Degree
AT5G15150	**HAT7**	**1**	303	185	19
AT3G24120	AT3G24120	2	225	NA	NA
AT1G26960	**HB-23**	**3**	223	484	3
AT5G65310	ATHB-5	4	218	64	49
AT1G33240	**GTL1**	**5**	207	396	5
AT1G13260	RAV1	6	189	116	31
AT1G69780	**HB-13**	**7**	183	NA	NA
AT3G01220	**HB-20**	**8**	181	NA	NA
AT4G31800	WRKY18	9	165	143	24
AT3G22830	HSFA6B	10	162	NA	NA

Fig. S18. Comparison of a CellOracle GRN from scRNA-seq to a BL GRN from bulk omics. (A) Top 100 transcription factors ranked by out-degree from the BL 2-hour elongating cortex CellOracle GRN inferred using scRNA-seq or a previously described GRN inferred from bulk data (*21*). **(B)** Comparison of transcription factor (TF) rank and out degree for the top 10 transcription factors in the BL 2 hour elongating cortex CellOracle GRN.

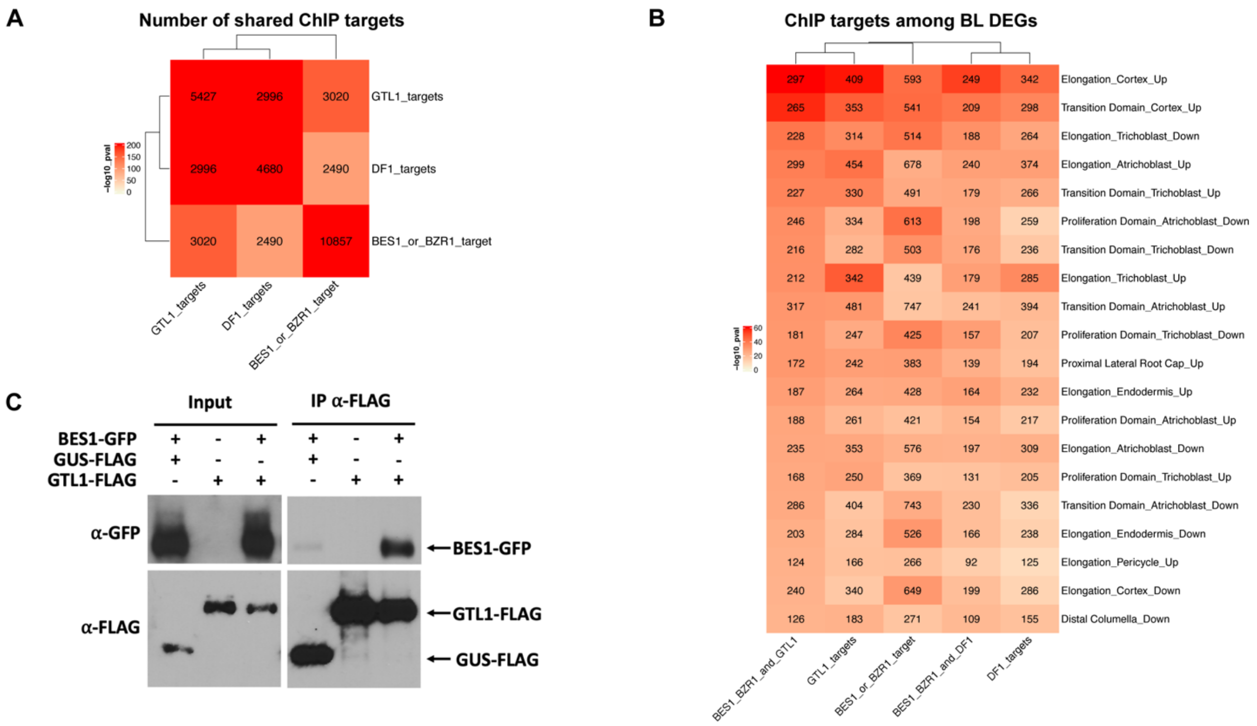

Fig. S19. BES1 and GTL1 interact and share a common set of target genes.

(A) Comparison of BES1 or BZR1, GTL1, and DF1 ChIP targets showing an overrepresentation of shared target genes. **(B)** Comparison of ChIP targets from (A) with BL 2 hour vs BRZ DEGs. The top 20 cell type/developmental stage combinations that are enriched for BES1 or BZR1 and GTL1 shared targets are shown. For (A) and (B) color represents −log10 p-values from the indicated overlaps calculated from Fisher's exact test by GeneOverlap. The number of genes in each intersection is indicated inside each box. **(C)** Co-Immunoprecipitation demonstrating BES1 interaction with GTL1. GTL1-FLAG immunoprecipitated with anti-FLAG beads pulled down BES1-GFP, whereas a GUS-FLAG negative control did not.

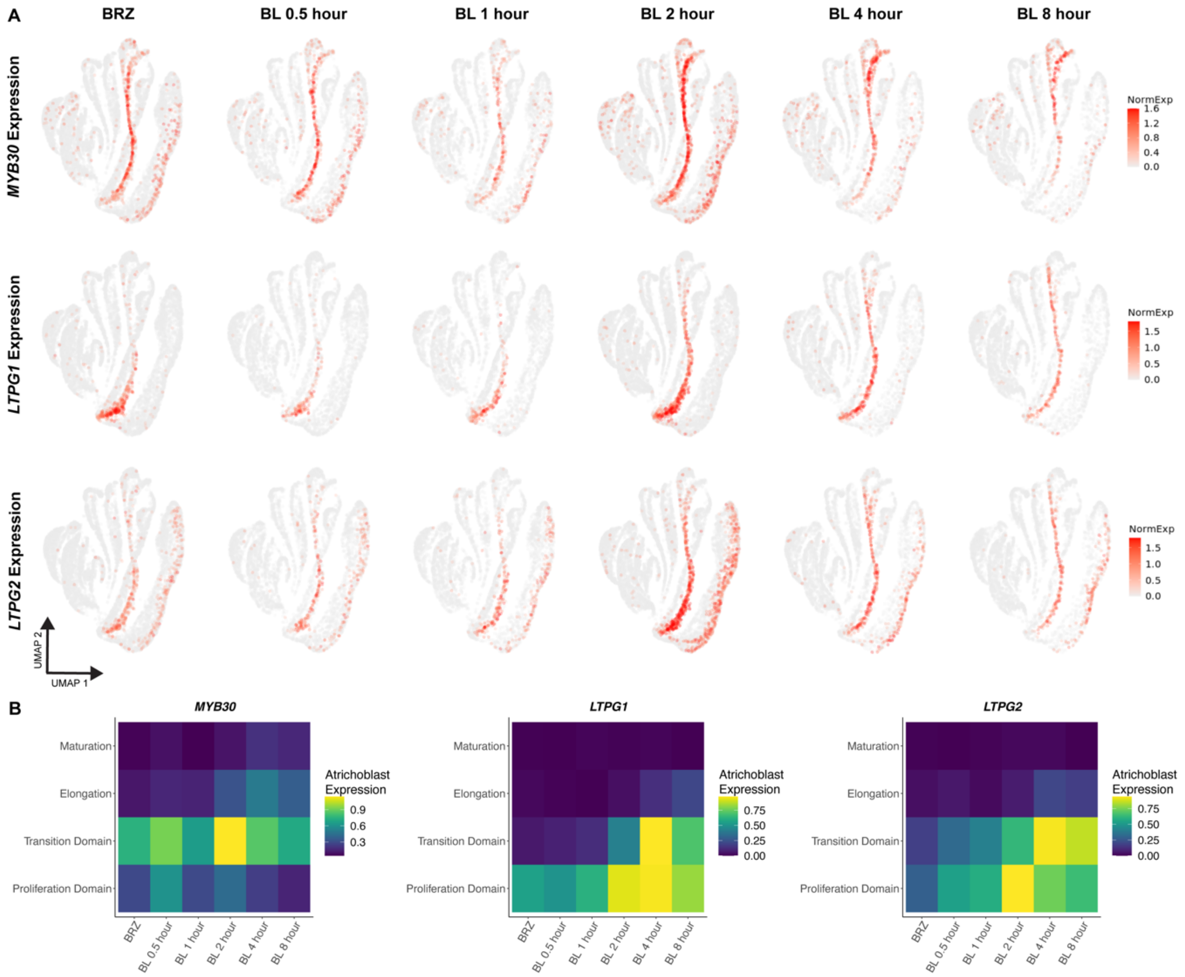

Fig. S20. ***MYB30*** **and its target genes** ***LTPG1*** **and** ***LTPG2*** **are induced in atrichoblasts of the BL scRNA-seq time series. (A)** UMAP projections showing expression levels of *MYB30*, *LTPG1,* or *LTPG2* over the brassinosteroid time series scRNA-seq experiment. The color scale represents log normalized, corrected UMI counts. **(B)** Gene expression trends for *MYB30*, *LTPG1,* or *LTPG2* along the developmental zones of atrichoblasts for each time point of the brassinosteroid time course. Color bar indicates the scaled expression level in atrichoblasts.

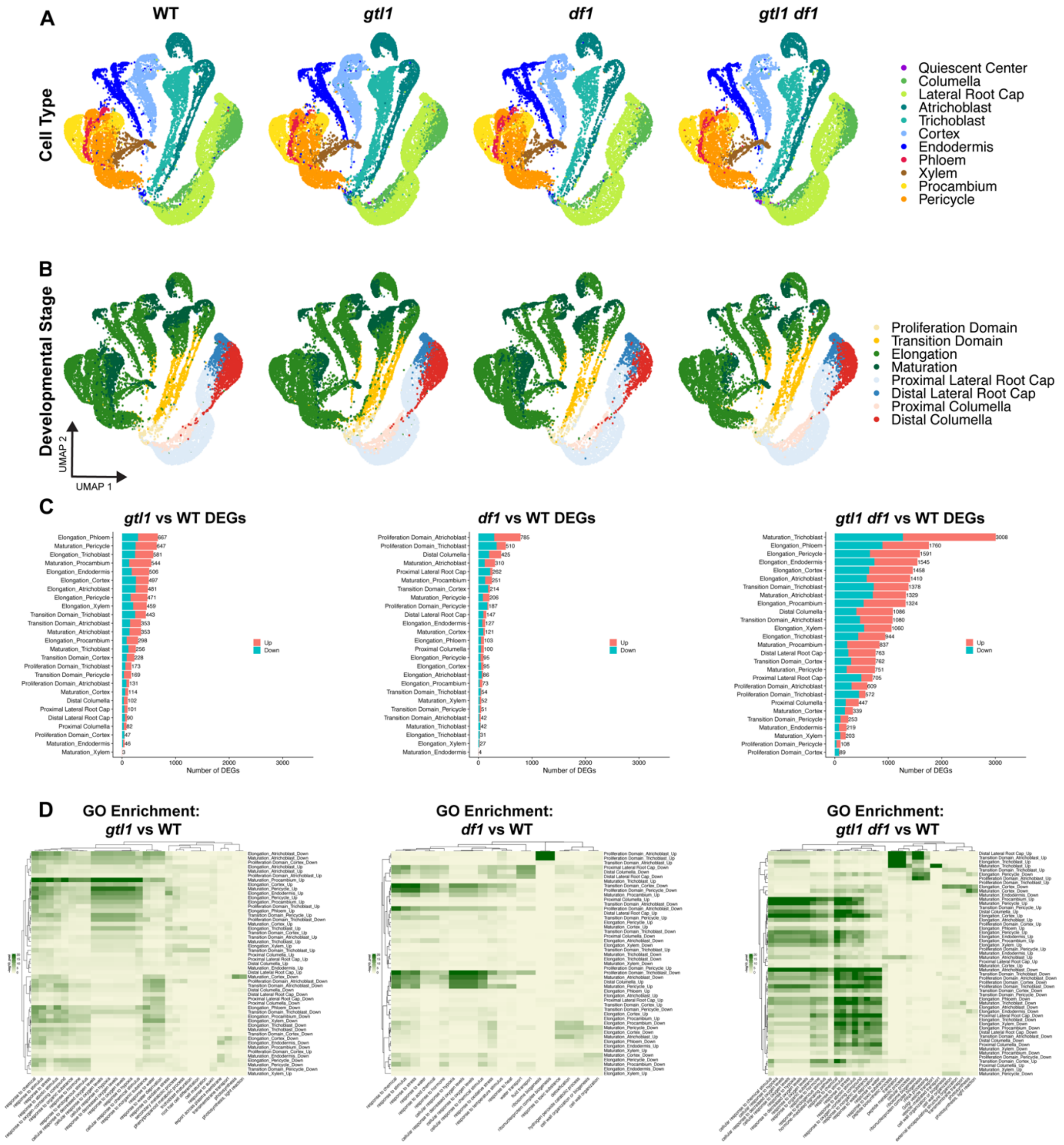

Fig. S21. Differential expression analysis from *gtl1*, *df1*, and *gtl1 df1* scRNA-seq.

(**A-B)** Two-dimensional uniform manifold approximation and projection (UMAP) embedding of 18,904 WT, 22,594 *gtl1*, 15,678 *df1,* and 17,634 *gtl1 df1* cells across 2 biological replicates of scRNA-seq. Colors indicate (A) cell type or (B) developmental stage annotation. **(C)** Number of DEGs for each cell type/developmental stage combination in *gtl1, df1,* or *gtl1 df1* scRNA-seq compared to WT. Color indicates the number of up-regulated vs down-regulated genes. **(D)** GO enrichment of DEGs in *gtl1, df1,* or *gtl1 df1* scRNA-seq compared to WT.

Movie S1.
Time-lapse confocal microscopy showing pC/VIF2-H2B-Venus in wild-type and gtl1 df1.

Data S1.
Summary of the scRNA-seq samples reported in this study.

Data S2.
Marker genes for updated developmental annotation of Shahan et al. WT root atlas.

Data S3.
DEGs from pseudobulk analysis of scRNA-seq datasets.

Data S4.
CellOracle GRN centrality metrics from brassinosteroid time series.

Data S5.
Predicted targets of HAT7 and GTL1 family transcription factors from elongating cortex CellOracle GRNs in brassinosteroid time series.

Data S6.
Oligos used in this study.
