# Supplementary material for: Brassinosteroid gene regulatory networks at cellular resolution in the Arabidopsis root
Source: Science. Author manuscript; Available in PMC 2023 Apr 21. (PMC10119888; doi:10.1126/science.adf4721)
Supplement: Data_S1 [file NIHMS1889089-supplement-Data_S1.pdf]

Data S1: Summary of the scRNA-seq samples reported in this study

Sample	Name	Genotype	Treatment	Date	10X_chemistry	Number of cells after COPILOT filtering	Number of genes detected	Median UMI counts per cell	Median number of genes detected per cell
sc_1	WT control	WT	Control	20191214	v3	9759	21844	3028	1528
sc_2	WT BRZ control	WT	BRZ	20191214	v3	7512	22403	6274	2497
sc_5	WT BRZ then 2 hour BL	WT	BL	20191214	v3	9790	22656	5415.5	2421.5
sc_43	WT BRZ control	WT	BRZ	20200212	v3	8874	22534	4936	2071
sc_44	WT BRZ then 30 mins BL	WT	BL	20200212	v3	6843	22046	6935	2441
sc_45	WT BRZ then 1 hour BL	WT	BL	20200212	v3	6336	22434	5525.5	2067
sc_46	WT BRZ then 2 hour BL	WT	BL	20200212	v3	6847	22490	7506	2632
sc_47	WT BRZ then 4 hour BL	WT	BL	20200212	v3	6907	22893	6430	2297
sc_48	WT BRZ then 8 hour BL	WT	BL	20200212	v3	6389	22873	5230	2014
sc_49	WT BRZ then 2 hour BL	WT	BL	20200212	v3	5638	22567	7764	2576
sc_50	WT BRZ control	WT	BRZ	20200212	v3	5087	22014	6800	2310
sc_111	WT control	WT	Control	20210621	v3.1	7449	24906	10146	2862
sc_112	WT BRZ	WT	BRZ	20210621	v3.1	6947	24851	8226	2611
sc_113	WT control	WT	Control	20210621	v3.1	9193	25398	8050	2788
sc_114	WT BRZ	WT	BRZ	20210621	v3.1	7373	25194	10426	3347
sc_122	WT	WT	Control	20210804	v3.1	11614	21930	6359	2377
sc_123	gtl1-1	gtl1	Control	20210804	v3.1	11141	22145	7475	2537
sc_124	df1-1	df1	Control	20210804	v3.1	9058	21751	6572.5	2275.5
sc_125	gtl1-1 df1-1	gtl1_df1	Control	20210804	v3.1	10012	22457	8517	2714
sc_126	WT	WT	Control	20210804	v3.1	7290	21885	9675	2822
sc_127	gtl1-1	gtl1	Control	20210804	v3.1	11453	22057	6277	2299
sc_128	df1-1	df1	Control	20210804	v3.1	6620	22152	11138.5	2978
sc_129	gtl1-1 df1-1	gtl1_df1	Control	20210804	v3.1	7622	22388	10281.5	3008
sc_130	WT	WT	Control	20211001	v3.1	6589	21554	10327	2983
sc_131	bri1-T	bri1_T	Control	20211001	v3.1	7621	22130	9461	3148
sc_133	pGL2:BRI1-GFP_bri1-T	pGL2_BRI1_GFP_bri1_T	Control	20211001	v3.1	5975	22518	11136	3370
sc_134	WT	WT	Control	20211001	v3.1	7745	22305	10538	3038
sc_135	bri1-T	bri1_T	Control	20211001	v3.1	5028	21909	11838	3550.5
sc_137	pGL2:BRI1-GFP_bri1-T	pGL2_BRI1_GFP_bri1_T	Control	20211001	v3.1	1903	21948	18497	4085
